# scCancer2: data-driven in-depth annotations of the tumor microenvironment at single-level resolution

**DOI:** 10.1093/bioinformatics/btae028

**Published:** 2024-01-18

**Authors:** Zeyu Chen, Yuxin Miao, Zhiyuan Tan, Qifan Hu, Yanhong Wu, Xinqi Li, Wenbo Guo, Jin Gu

**Affiliations:** MOE Key Laboratory of Bioinformatics, BNRIST Bioinformatics Division, Institute for Precision Medicine & Department of Automation, Tsinghua University, Beijing 100084, China; MOE Key Laboratory of Bioinformatics, BNRIST Bioinformatics Division, Institute for Precision Medicine & Department of Automation, Tsinghua University, Beijing 100084, China; Department of Finance, Shanghai Advanced Institute of Finance, Shanghai Jiao Tong University, Shanghai 200240, China; MOE Key Laboratory of Bioinformatics, BNRIST Bioinformatics Division, Institute for Precision Medicine & Department of Automation, Tsinghua University, Beijing 100084, China; MOE Key Laboratory of Bioinformatics, BNRIST Bioinformatics Division, Institute for Precision Medicine & Department of Automation, Tsinghua University, Beijing 100084, China; MOE Key Laboratory of Bioinformatics, BNRIST Bioinformatics Division, Institute for Precision Medicine & Department of Automation, Tsinghua University, Beijing 100084, China; MOE Key Laboratory of Bioinformatics, BNRIST Bioinformatics Division, Institute for Precision Medicine & Department of Automation, Tsinghua University, Beijing 100084, China; MOE Key Laboratory of Bioinformatics, BNRIST Bioinformatics Division, Institute for Precision Medicine & Department of Automation, Tsinghua University, Beijing 100084, China

## Abstract

**Summary:**

Single-cell RNA-seq (scRNA-seq) is a powerful technique for decoding the complex cellular compositions in the tumor microenvironment (TME). As previous studies have defined many meaningful cell subtypes in several tumor types, there is a great need to computationally transfer these labels to new datasets. Also, different studies used different approaches or criteria to define the cell subtypes for the same major cell lineages. The relationships between the cell subtypes defined in different studies should be carefully evaluated. In this updated package scCancer2, designed for integrative tumor scRNA-seq data analysis, we developed a supervised machine learning framework to annotate TME cells with annotated cell subtypes from 15 scRNA-seq datasets with 594 samples in total. Based on the trained classifiers, we quantitatively constructed the similarity maps between the cell subtypes defined in different references by testing on all the 15 datasets. Secondly, to improve the identification of malignant cells, we designed a classifier by integrating large-scale pan-cancer TCGA bulk gene expression datasets and scRNA-seq datasets (10 cancer types, 175 samples, 663 857 cells). This classifier shows robust performances when no internal confidential reference cells are available. Thirdly, scCancer2 integrated a module to process the spatial transcriptomic data and analyze the spatial features of TME.

**Availability and implementation:**

The package and user documentation are available at http://lifeome.net/software/sccancer2/ and https://doi.org/10.5281/zenodo.10477296.

## 1 Introduction

The rapid development of single-cell RNA-sequencing (scRNA-seq) and spatial transcriptome (ST) technologies has promoted the accumulation of large-scale single-cell resolution omics data, enabling us to analyze the composition of tumor microenvironment (TME) more precisely and comprehensively.

Currently, researchers have defined many meaningful TME cell subtypes in various cancer types (Zheng *et al.* 2017, Guo *et al.* 2018, Zhang *et al.* 2018), which provides the opportunity to train supervised machine learning models and transfer these expert annotations to new datasets. Therefore, we upgraded our toolkit scCancer (Guo *et al.* 2021) to a new version (scCancer2) and improved the cell type annotation to more subtle subtypes. The updated version tended to preserve the labeling information or prior knowledge in the original references and assigned multiple labels to the user’s data with a supervised machine learning framework. Compared with previous methods for cell type annotation (Kiselev *et al.* 2018, Alquicira-Hernandez *et al.* 2019, Aran *et al.* 2019, de Kanter *et al.* 2019, Pliner *et al.* 2019, Tan and Cahan 2019, Li *et al.* 2020, Galdos *et al.* 2022), we focused on the scenarios of TME and provided a set of classifiers for annotating the cell types and subtypes defined in different references. As the accumulation of scRNA-seq based TME studies, there is a common need to examine the differences and similarities of the cell subtypes defined in different references. So, based on the trained classifiers, we established a cell subtype similarity map across multiple references by comparing the predicted labels on large-scale tumor single-cell collections (15 datasets, 594 samples, and 1 213 469 cells; 5 training datasets for T cell subtypes, 3 for B cells, 6 for myeloid cells, 4 for endothelial cells, and 4 for fibroblasts). The generated similarity map is a meaningful source to summarize and compare the abundant knowledge from different datasets.

At the single-cell level, in addition to cell subtype annotation, it is also crucial to identify the malignant cells in TME. Currently, the methods based on copy number variations (CNV) (Patel *et al.* 2014, Gao *et al.* 2021, Guo *et al.* 2021) have been applied to the malignancy annotation in various cancer types. However, when no internal confidential reference cells are available, CNV-based methods frequently get unstable results. To be compatible with these special scenarios, we developed an additional data-driven method. Firstly, a reference dataset combining scRNA-seq dataset (663 857 cells in 175 samples) and bulk RNA-seq data (7012 TCGA samples) was established across multiple cancer types and then an XGBoost (Chen and Guestrin 2016) based classifier was trained to identify malignant cells with high generalization ability. Besides, the classifier achieved higher computational efficiency and lower memory burden, which is suitable for processing large-scale datasets. To demonstrate the robustness of this module in scCancer2, we categorized the test samples into four groups, including tumor samples with the bimodal or unimodal distribution of malignancy score, normal samples, and organoid samples. Extensive tests proved that it can be a great supplement to CNV-based methods.

Finally, the spatial dimension is also highly significant in characterizing the TME. To systematically dissect the TME spatial features, we constructed an automated ST analysis module, which includes three analytical perspectives: spatial interaction, spatial heterogeneity, and spatial structure. By applying it to 60 samples from various cancer types, we demonstrated its good performance.

Overall, taking advantage of the accumulated scRNA-seq data of cancer clinical samples, the in-depth expert annotations for TME in each study, and the new spatial information brought by the ST technology, scCancer2 improved its functionalities for automatically dissecting the complex TME features. The performances of scCancer2 have been extensively tested on a large amount of data across various cancer types.

## 2 Materials and methods

### 2.1 TME cell subtype annotation

#### 2.1.1 Cell subtype annotation method within a single dataset

Firstly, we divided the dataset into training and validation sets in a 4:1 ratio (5-fold cross-validation, stratified sampling by cell subtype). Given a training expression matrix *X* of cell subtype labels *L* and the marker genes of every subtype $G_{\mathrm{prior}}$ (or differentially expressed genes of clusters), we first selected representative cells to construct core training dataset according to the marker score generated from the Garnett (Pliner *et al.* 2019) method. The “Aggregate Marker Score” function evaluated the expression of important features within cells, and we selected samples *C* that express specific cell type features as core training dataset. Then, the Entropy test (Li *et al.* 2020) or Highly Regional Genes (Wu *et al.* 2022a) was used for feature selection. The genes *G* used for training were the union of input markers $G_{\mathrm{prior}}$ and genes selected from the scRNA-seq expression matrix.

Therefore, we obtained an expression matrix $X_{C,G}$ for model training. We trained a multinomial model in which every parameter represents the expression probability *P* of a corresponding gene. For the validation set, we also evaluated the expression of marker genes. We labeled a cell as “unknown” when it did not express any marker gene in the prior information, which means that for every possible cell subtype, the “Aggregate Marker Score” function outputs a low marker score.

Then, we assigned cell subtypes to other unlabeled cells with maximum likelihood estimation (MLE). *P* was weighted by the marker score mentioned above. For every possible cell subtype *j*, gene *i*, and gene expression vector *x*, we assigned a cell label with MLE as follows:

(1) $$\begin{array}{c} w_{i}=1+I(i\in G_{\mathrm{prior}})\;\text{log}(1+{\text{score}}_{i})\\ \hat{j}={\text{argmax}}_{j}P(x|j)\\ ={\text{argmax}}_{j}\;\text{log}\;\left(\prod_{i}{\left(w_{i}p_{ij}\right)}^{x_{i}}\right)\\ ={\text{argmax}}_{j}\left(\sum_{i}x_{i}\;\text{log}\;\left(w_{i}p_{ij}\right)\right) \end{array}$$

We evaluated the performance of our model through 5-fold cross-validation on 15 published datasets, using the mean and variance of classification accuracy and kappa index as indicators. We finally obtained 22 cell subtype templates across five major cell types in total (Supplementary Table S2). In addition, we compared scCancer2 with scCancer(Guo *et al.* 2021), Scibet (Li *et al.* 2020), scPred (SVM) (Alquicira-Hernandez *et al.* 2019), CHETAH (de Kanter *et al.* 2019), scmap (Kiselev *et al.* 2018), SingleCellNet (Tan and Cahan 2019), and XGBoost (Chen and Guestrin 2016) on six datasets with five major cell types and three sequencing technologies.

#### 2.1.2 Multi-label annotation structure

For the newly input dataset *D*, we first annotated the cell types with scCancer (Guo *et al.* 2021) and split the dataset based on major cell types. For every subset $D_{\mathrm{sub}}$, we traversed all corresponding cell subtype templates and annotated cell subtypes with the above pipeline iteratively. For each reference dataset, as we have trained five “weak” models during cross-validation, we integrated the prediction of the models through voting. Cells without any predicted label with a vote count >2 (≥3) were also labeled as “unknown.” The output is summarized in Supplementary Table S3.

#### 2.1.3 Similarity map of different subtype labels

Defining a set $C_{ij}$ as cells assigned to the *j*th subtype in the *i*th group of labels, namely, the *i*th dataset, and the similarity matrix as *S*, the similarity of every two labels was calculated as follows:

(2) $$S[\mathrm{index}1,\mathrm{index}2]=||C_{ij}\cap C_{mn}||/||C_{ij}\cup C_{mn}||$$

where the index is the position of a subtype after flattening all the subtype labels into a vector, and the dimension of matrix *S* is the length of the label vector, namely, the total number of subtype labels. Finally, we visualized the similarity matrix by heatmap, hierarchical clustering tree, and multi-dimensional scaling in R packages. Cross-annotation process within the training set is summarized in Algorithm 1. Only the requirement changes for the new test dataset: 1 test dataset, *N* annotation models, and *N* groups of labels.

Algorithm 1. Multi-model and multi-dataset cross annotation

**Require:** *N* reference datasets (*D*); *N* groups of labels (*L*); *N* annotation models (*M*).

 **for**i=1 to *N* **do**

  **for**j=1 to *N* **do**

   Test model $M_{j}$ on dataset $D_{i}$, assign cell subtypes.

  **end for**

  Define: $L_{im}\leftarrow m^{th}\;\mathrm{subtype}\;in\;L_{i},L_{jn}\leftarrow n^{th}\;\mathrm{subtype}\;in\;L_{j}$

 Obtain: $C_{im}=\{\mathrm{cell}|\mathrm{cells}\;\mathrm{assigned}\;to\;L_{im}\},C_{jn}=\{\mathrm{cell}|\mathrm{cells}\;\mathrm{assigned}\;to\;L_{JN}\}$

   Jacobian similarity matrix: $J_{\mathrm{row},\mathrm{col}}=\frac{\left|C_{im}\;\cap \;C_{jn}\right|}{\left|C_{im}\;\cup \;C_{jn}\right|}$

 **end for**

 Integrate *N* matrixes with mean and variation.

 $J\leftarrow \mathrm{Mean}(J_{1},J_{2},\ldots ,J_{N})$

 $V\leftarrow \mathrm{Var}(J_{1},J_{2},\ldots ,J_{N})$

 H←hclust(J)

 M←cmdscale(J)

**Ensure:** Jacobian similarity matrix of all labels (*J*); variance across different datasets (*V*); hierarchical clustering results (*H*); MDS coordinates (*M*).

### 2.2 Malignant cell identification

#### 2.2.1 Data collection, preprocessing, and annotation

We first collected 49 tumor samples sequenced by 10X Genomics with no previous malignant annotation. We calculated the copy number variation score of tumor samples through the inferCNV (Patel *et al.* 2014) and plot the distribution of malignancy scores. We retained 18 samples with an obvious bimodal distribution.

Although inferCNV has been proven to be reliable when the malignancy score follows a bimodal distribution, we found that epithelial cells were highly correlated with malignancy in the labeled results. To prevent the model from regarding the features of epithelial cells as malignant, we collected nine normal samples with more normal epithelial cells. Additionally, we downloaded and integrated published datasets from the TISCH2 database (Han *et al.* 2022) as a supplement. (Supplementary Table S4)

To integrate samples from different sources, we preprocessed the data through scanpy (Wolf *et al.* 2018) and concatenated the expression matrices based on common genes. The combined scRNA-seq dataset was an expression matrix with 466 468 cells and 7749 genes.

#### 2.2.2 Feature selection combining bulk RNA-seq and scRNA-seq

We first collected bulk RNA-seq data of 14 cancer types from TCGA. Then we pre-processed and integrated the samples based on common genes, obtaining an expression matrix with 7012 samples and 31 531 genes (Supplementary Table S5).

We calculated the top 5000 differentially expressed genes between malignant samples and normal samples across 14 cancer types using the edgeR (Robinson *et al.* 2010) package. From these 14 groups of differentially expressed genes, we identified a total of 1997 high-frequency genes that appeared >8 times, of which 1069 appeared in the integrated single-cell dataset, named as $G_{\mathrm{bulkDE}}$.

As for scRNA-seq data, we selected 2000 genes with the highest expression variance as $G_{\mathrm{scHV}}$. The features selected from scRNA-seq data $G_{\mathrm{scRNA}}$ for model training were the union of $G_{\mathrm{bulkDE}}$ and $G_{\mathrm{scHV}}$. Finally, the obtained reference dataset was an expression matrix with 466 468 cells and 2756 genes.

#### 2.2.3 Model training and performance evaluation

We divided the training set and validation set by sample sources (Supplementary Table S4) and trained an XGBoost model with a binary logistic objective function. Confusion matrix, prediction accuracy, and AUC score were calculated through scikit-learn (Pedregosa *et al.* 2011) for quantitative representation, and UMAP was applied to qualitatively demonstrate the aggregation of labels on the 2D space.

We analyzed the performance of scCancer2 under various scenarios: tumor samples with either bimodal or unimodal distribution of malignancy scores, normal samples, and organoid samples. Additionally, we evaluated the generalization ability of scCancer2 by changing the cancer types in the training set or testing it on new cancer types.

### 2.3 ST analysis

#### 2.3.1 Data collection

The test datasets for the spatial module in scCancer2 were obtained from previous studies and 10X Genomics. We collected 60 samples across nine cancer types in total (Supplementary Table S6).

#### 2.3.2 Spatial interaction analysis

First, we automatically identified boundary regions between two clusters. All spots belonging to certain two clusters are extracted. A spot of one cluster is considered to be in the boundary area of these two clusters if there are spots of the other cluster in its six neighbors. Every spot in these two clusters is examined. We identify numerous boundary regions comprising connected regions formed by all boundary points. Furthermore, boundary regions with fewer than 20 spots were removed to provide sufficient data for *P*-value calculations.

Then we evaluated the interaction strength and *P*-value at the boundary regions. We extracted expressions of the approximately 1400 known L–R pairs from the CellPhoneDB dataset (Efremova *et al.* 2020). The L–R interaction strength between two clusters is defined as the average expression of the ligand and receptor in each cluster, respectively. To identify significant interactions, we randomly disrupted the labels of spots 1000 times. Each time, we calculated the strength of every interaction pair within each group using the new labels, forming a null distribution consisting of 1000 sampling points for each pair. *P*-values were obtained by calculating the proportion of the strengths which are more than the actual strength. We then filtered out weak L–R pairs based on their strengths and *P*-values, which were calculated from the null distribution mentioned above. Finally, we visualized these significant interactions between clusters through a scatter plot.

#### 2.3.3 Phenotypic heterogeneity analysis

We mainly considered 14 phenotypic characteristics from CancerSEA (Yuan *et al.* 2019). The average expression of signature genes and the “AddModuleScore” function in Seurat are both optional to calculate phenotypic scores. To demonstrate the phenotypic differences in different regions of tumor tissue, we manually divided the HCC-1L sample into three regions (tumor, capsule, and normal) based on the clustering results and the H&E image. We derived the phenotypic scores of three regions and used the Wilcox test to determine the significance of pairwise differences.

#### 2.3.4 Spatial structure detection

Firstly, we scored the enrichment of corresponding gene signatures with average expression or “AddModuleScore” in Seurat. The Global Moran’s I (GMI) was then calculated based on signature scores to infer the existence of corresponding spatial structures. GMI was calculated as follows, where *x* is the signature score of each spot, *n* is the number of spots, and $w_{ij}$ is the weight of spot *i* and spot *j*, $d_{ij}$ is the distance of spot i and spot j, min(d) is the minimum distance of each pair of spots.

(3) $$\begin{array}{c} \mathrm{GMI}=\frac{n}{S_{0}}\frac{\sum_{i=1}^{n}\sum_{j=1}^{n}w_{ij}(x_{i}-\bar{x})(x_{j}-\bar{x})}{\sum_{i=1}^{n}{\left(x_{i}-\bar{x}\right)}^{2}}\\ S_{0}=\sum_{i=1}^{n}\sum_{j=1}^{n}w_{ij}\\ w_{ij}=\{\begin{array}{cc} \frac{1}{d_{ij}^{2}}, & d_{ij}<2.5*\min(d)\\ 0, & d_{ij}\geq 2.5*\min(d) \end{array} \end{array}$$

A positive GMI value indicates a tendency toward clustering, and the closer GMI is to 1, the more significant the clustering is. It is assumed as a default that samples exhibiting a GMI >0.5 possess distinct spatial structures. Then we calculated the Local Moran’s I (LMI) (Anselin 1995) to determine the position of spatial structures:

(4) $$\begin{array}{c} LMI_{i}=\frac{x_{i}-\bar{x}}{S_{i}^{2}}\sum_{j=1,j\neq i}^{n}w_{ij}(x_{j}-\bar{x})\\ S_{i}^{2}=\frac{\sum_{j=1,j\neq i}^{n}{\left(x_{j}-\bar{x}\right)}^{2}}{n-1} \end{array}$$

A positive value for LMI indicates that the score has neighboring scores with similarly high or low attribute values. Since LMI is a relative measure, we calculated the z-scores to interpret it and obtained *P*-values:

(5) $$\begin{array}{c} z_{i}=\frac{LMI_{i}-E[LMI_{i}]}{\sqrt{\mathrm{Var}[LMI_{i}]}} \end{array}$$

Furthermore, we used two strategies to overcome the high false positive rate of the LMI-based method. The first one is *P*-value correction. FDR correction was implemented to adjust the *P*-values obtained from the z-scores. A spot is determined as part of a specific structure if its: (i) adjusted *P*-value < .05; and (ii) signature score exceeds the median. The other one is based on *z*-scores. We recalculated the *z*-scores of signature scores in areas with *P*-values <.05 and subsequently obtained the corresponding new *P*-values. Spots with new *P*-values <.05 were considered regions containing spatial structures.

#### 2.3.5 Analysis of tertiary lymphoid structures and tumor capsules

We collected the TLS-50 signature from Wu *et al.* (2021a) and calculated the average expression of these 50 signatures in each spot as TLS scores. TLS areas were obtained using the method mentioned in 4.3 and we removed false positive results based on *z*-scores. Next, we combined the tumor samples from Wu *et al.* (2021a, b, 2022b) with common genes and obtained a matrix containing 821 TLS spots. Then, we analyzed the cellular composition of the detected TLS spots with “FindTransferAnchors” and “TransferData” in Seurat (Hao *et al.* 2021). We chose a multi-modal PBMC dataset in Hao *et al.* (2021) as the reference and mapped the cell annotations to the combined TLS spots. To save memory, we extracted 50% of the original dataset by cell subtypes with the stratified sampling method.

To obtain the signatures of tumor capsules, we clustered the “HCC-1L” sample in Wu *et al.* (2021a), and used “FindMarkers” in Seurat to obtain the top 50 differentially expressed genes of the tumor capsule cluster (Capsule-50). Capsule scores were calculated using the average expression of Capsule-50. Tumor capsule areas were obtained using the method mentioned in Section 2.3 and we removed false positive results based on adjusted *P*-values.

## 3 Results

### 3.1 Overview of scCancer2

**Figure 1.**
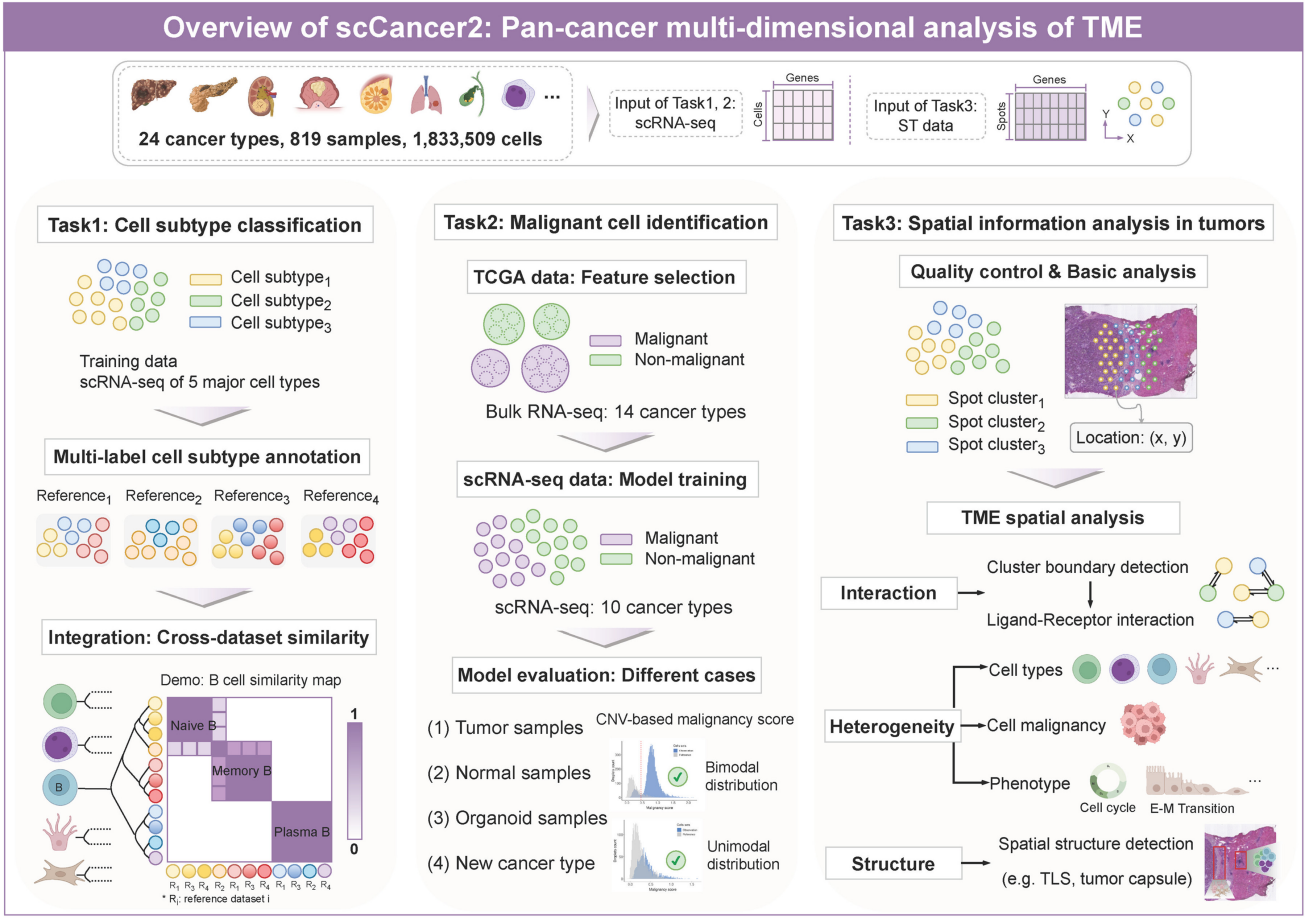
The overview of scCancer2.

We implemented three new modules in scCancer2 (Fig. 1). Firstly, scCancer2 automatically performed cell subtype classification with built-in models and output multi-label annotations. To visualize the relationship of labels quantitatively, a similarity map of cell subtypes from different atlases was calculated for every major cell type. Secondly, scCancer2 identified malignant cells based on bulk RNA-seq and scRNA-seq with the XGBoost. Our method can be directly applied to various types of samples without relying on the inference of CNVs. Thirdly, scCancer2 performed systematical analyses of spatial transcriptomics data for cancer research, mainly consisting of basic analysis and TME spatial analysis. The information on packages and methods newly integrated into scCancer2 were summarized in Supplementary Table S1.

### 3.2 scCancer2 upgrades TME cell type annotation to the subtype level

Advances in scRNA-seq enabled researchers to provide accurate depictions of TME. The cell annotation of recently published datasets was usually precise to the cell subtype level, which can serve as reference datasets for cell subtype classification. We collected and preprocessed a total of 15 published datasets across 17 human cancer types and three sequencing technologies (10X Genomics, Smart-seq2, InDrop). There are 594 samples with 1 213 469 cells in total (Table 1).

**Table 1.** The list of scRNA-seq datasets used in cell subtype annotation.

Accession	Organ	No. of cells^a^	No. of samples	Cell type^b^	Technology
GSE98638 (Zheng *et al.* 2017)	Liver	5063	6	T cell	Smart-Seq2
GSE99254 (Guo *et al.* 2018)	Lung	12 346	14	T cell	Smart-Seq2
GSE108989 (Zhang *et al.* 2018)	Colorectal	11 138	12	T cell	Smart-Seq2
GSE178341 (Pelka *et al.* 2021)	Colorectal	371 223	181	All	10X
GSE176078 (Wu *et al.* 2021 b)	Breast	100 064	26	All	10X
GSE154763 (Cheng *et al.* 2021)	Pan-cancer	65 083	48	Myeloid cell	10X
GSE127465 (Zilionis *et al.* 2019)	Lung	54 773	40	Immune cell	inDrop
GSE150430 (Chen *et al.* 2020)	Nasopharyngeal	48 584	16	Immune cell	10X
GSE146771 (Zhang *et al.* 2020)	Colorectal	54 285	20	All	Smart-Seq2, 10X
GSE140228 (Zhang *et al.* 2019)	Liver	73 286	41	Immune cell	Smart-Seq2, 10X
GSE103322 (Puram *et al.* 2017)	Head and Neck	5902	18	All	Smart-Seq2
GSE132465 (Lee *et al.* 2020)	Colorectal	65 362	33	All	10X
GSE131907 (Kim *et al.* 2020)	Lung	208 506	58	All	10X
E-MTAB-6149 (Lambrechts *et al.* 2018)	Lung	40 250	19	Stromal cell	10X
GSE156728^c^ (Zheng *et al.* 2021)	Pan-cancer	97 604	62	T cell	10X

a In the column “No. of cells,” the numbers represent the total number of cells in the datasets. For model training and validation, we usually selected specific cell types (a subset of the original dataset).

b In the column “Cell type,” “All” represents five major cell types: immune cells (T cells, B cells, and myeloid cells) and stromal cells (endothelial cells and fibroblasts). Epithelial cells are excluded.

c GSE156728 was treated as the query dataset. The patient samples were not used for model training.

Here, we implemented a rapid, supervised annotation framework integrating prior information. The training step for every single dataset mainly includes the following steps. Firstly, select cells by the expression of marker genes to construct high-quality training sets (Pliner *et al.* 2019). Secondly, select genes according to the statistic entropy (Li *et al.* 2020). Thirdly, train a multinomial model for each cell subtype and estimate the expression probability of the selected genes by maximum likelihood estimation (Section 2.1). Then, for each major cell type, we trained a series of subtype annotation models based on multiple reference datasets (Section 2.1).

**Figure 2.**
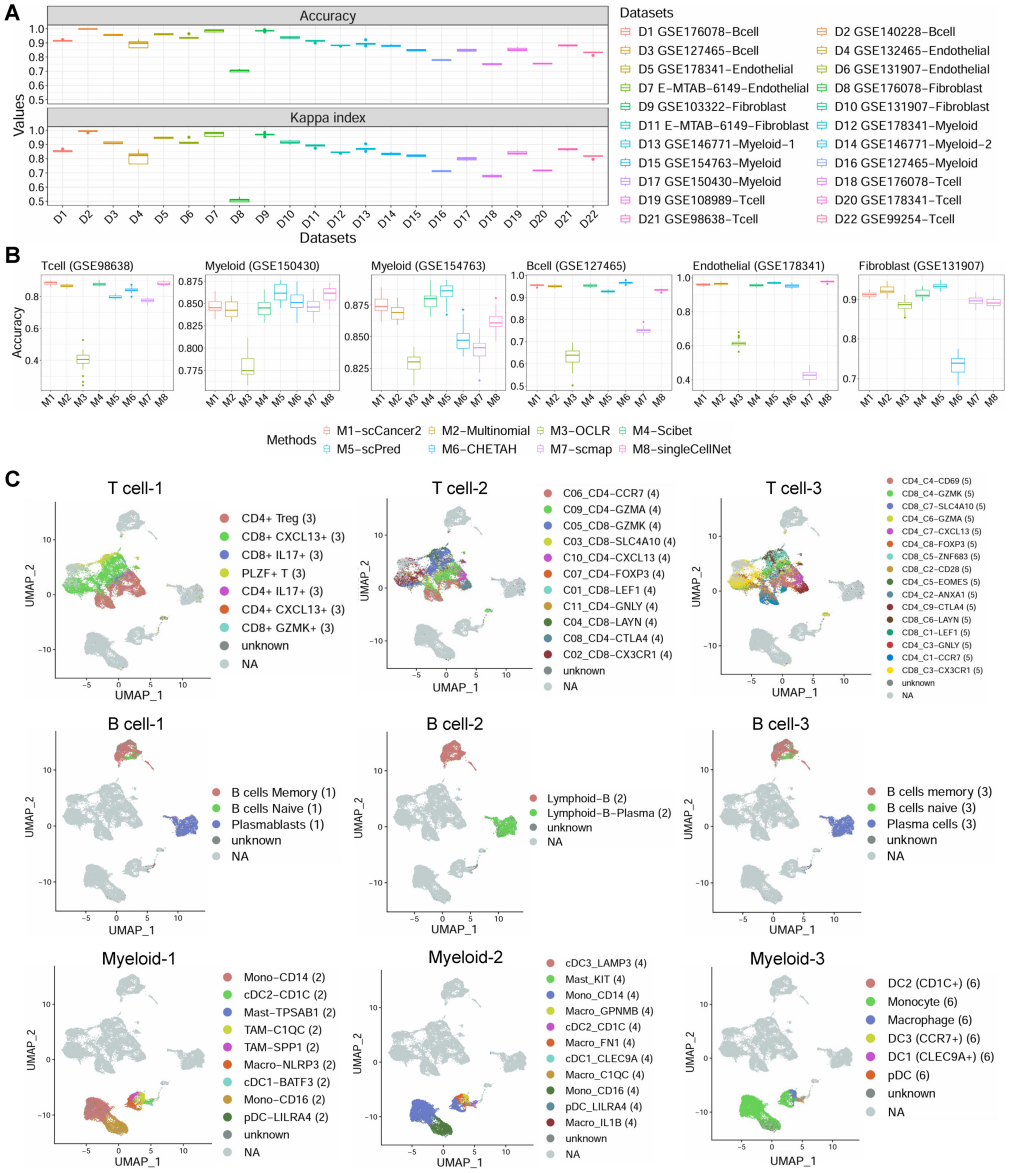
scCancer2 annotates cell subtypes of TME. (A) Performance evaluation of scCancer2 on cell subtype annotation task. The accuracy and kappa index were obtained by 5-fold cross-validation (stratified sampling by cell subtype). (B) Comparison of different machine learning methods on cell subtype annotation task. The results were obtained by 5-fold cross-validation. (C) Multi label annotation results on CRC example (immune cells). Rough cell labels (T cell, myeloid cell, B cell) were assigned to CRC example through OCLR in scCancer. The dataset was then divided by major cell types. Cell subtype annotation was achieved by scCancer2, forming a multi-model hierarchical cell type annotation. In each subfigure, the numbers after the label represent the literature serial number corresponding to the major cell type.

By applying to 594 samples in Table 1, we proved that scCancer2 had great generalization ability on TME datasets of five major cell types (Fig. 2A), including T cells, B cells, myeloid cells, endothelial cells, and fibroblasts. Moreover, we compared the framework with other benchmarks including scPred (Alquicira-Hernandez *et al.* 2019), one-class logistic regression (OCLR) (SOKOLOV *et al.* 2016), multinomial, Scibet (Li *et al.* 2020), CHETAH (de Kanter *et al.* 2019), scmap (Kiselev *et al.* 2018), and SingleCellNet (Tan and Cahan 2019) on six datasets containing five major cell types, and three sequencing technologies. Considering the prediction accuracy, computational efficiency, and model complexity, the results in Fig. 2B further indicate the reliability of our framework. Taking 43 817 immune cells in the colorectal cancer (CRC) dataset GSE146771 (Zhang *et al.* 2020) as an example, cell types were annotated hierarchically. We annotated major immune cell types through OCLR in scCancer (Guo *et al.* 2021). Then, for each major cell type, we assigned multiple sets of cell subtype labels based on multiple trained models in scCancer2 and visualized them respectively (Fig. 2C). Correspondence between the suffix number of labels and reference datasets can be found in Supplementary Table S2.

In summary, we fully preserved the original annotation from different cell atlases and transferred them to new datasets with a supervised machine-learning framework.

### 3.3 scCancer2 quantitatively evaluates the similarity of cell subtypes across datasets

Through the above process, scCancer2 outputs multi-label annotations where each cell is assigned multiple possible labels, retaining the original annotation in cell atlases. However, the cellular composition of different cancer types varied greatly. Considerable variations exist in expert annotations at the subtype level across different datasets. Therefore, depicting the relationship between cell subtypes defined in different studies is urgently needed.

We carried out a method to integrate the cell annotations from different experts. We annotated cell subtypes across datasets with those built-in models respectively and each label was assigned to several cells. Then, we extracted “label-barcode” sets from the results and derived the Jacobian similarity. Finally, the similarity matrix was visualized by heatmap, hierarchical clustering, and multi-dimensional scaling (Section 2.1). By observing the aggregation of labels in 2D space, we can understand the complicated relationship between different cell subtypes.

**Figure 3.**
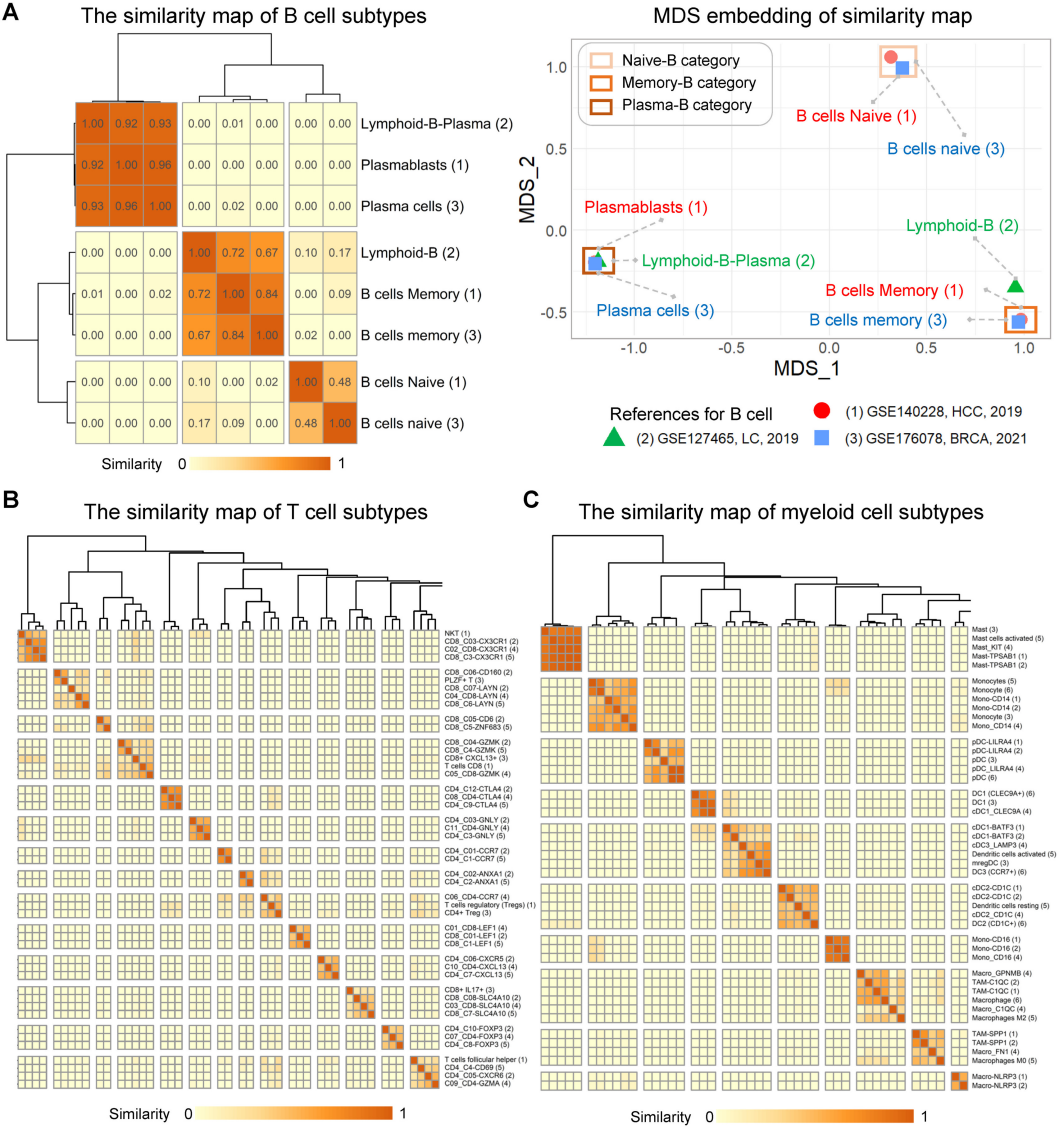
Generation of cross-dataset cell subtype similarity by scCancer2. (A) The cross-dataset similarity map of lymphoid B cell subtypes on CRC-example. The left panel is the heatmap of the similarity matrix. The right panel is the multi-dimensional scaling plot of the matrix, demonstrating the similarity of different labels. Color represents the source of subtype labels. (B) The cross-dataset similarity map of lymphoid T cell subtypes on CRC-example. (C) The cross-dataset similarity map of myeloid cell subtypes on CRC-example. * In each subfigure, the numbers after the label represent the literature serial number corresponding to the major cell lineage. The horizontal and vertical coordinates of the heatmap are the same. Each point in the matrix represents the Jaccord similarity between the horizontal axis label and the vertical axis label, and the diagonal element is 1. (B) and (C) are part of the similarity map (See Supplementary Figs S2–S6 and Supplementary Table S2 for full version and references).

Take CRC dataset (Zhang *et al.* 2020) as an example, cell annotation was conducted based on multiple trained models, and the cross-dataset label relationships of B cells were calculated, as shown in Fig. 3A. It was observed that the aggregation pattern of B cell labels was consistent with expectations: two naive B cell labels, three plasma cell labels, and two memory B cell labels showed a high degree of similarity. In particular, the “Lymphoid-B” in hepatocellular carcinoma (HCC) was expected to be the union of naïve B cells and memory B cells in non-small-cell lung cancer (NSCLC) and breast cancer (BRCA). Respectively, its similarity with the naive B cells was 0.10 and 0.17, and that with the memory B cells was 0.72 and 0.67 (Fig. 3A). The results indicate the inclusion relationship between these groups of labels, which is consistent with the biological meaning of the labels. In the right panel of Fig. 3A, subtypes of B cells can be clearly divided into three categories based on spatial distance. The “Lymphoid-B” label was located between the naive B category and the memory B category and closer to the latter.

Similarly, the similarity maps of T cells and myeloid cells were generated. We took a portion of the complete similarity graph in Fig. 3B and C. Firstly, cell subtypes from different studies of the same group of researchers were often named in a similar pattern. They were almost all clustered into the same category in the similarity map. For example, “CD4_C12-CTLA4,” “C08_CD4-CTLA4,” and “CD4_C9-CTLA4” in Fig. 3B. Except for identical labels mentioned above, cell subtypes named by different researchers with similar functions also showed high similarity in the heatmap. For example, “Mast,” “Mast cells activated,” “Mast_KIT,” and “MAST-TPSAB1” in Fig. 3C. Finally, we integrated the original annotations of the pan-cancer query dataset (Zheng *et al.* 2021) into the similarity graph. Observing the results in Supplementary Fig. S1, we found that the annotations of scCancer2 had a clear correspondence with the original expert annotations. Almost all newly assigned labels aggregated in the heatmap had corresponding original annotations. These results indicate that scCancer2 successfully depicted the cross-dataset relationship of complicated immune cell subtypes with similar biological functions (See Supplementary Figs S2–S6 for all results and the corresponding studies).

In summary, we quantitatively depicted the connections between cell subtypes from different cell atlases with the label similarity map. We extracted and summarized the abundant information in the multi-label annotation, which can be an important reference and literature indexing tool.

### 3.4 scCancer2 identifies malignant cells across multiple cancer types without internal references

Determining the malignancy of the cells is an important and challenging task, as it holds key significance not only for the subsequent analysis of tumor heterogeneity and microenvironmental characteristics but also for investigating the mechanisms of tumor occurrence and development. In scCancer (Guo *et al.* 2021), the cell malignancy scores were estimated based on inferCNV (Patel *et al.* 2014), and the malignancy labels were determined according to the bimodality observation of the scores after neighbor smoothing. However, CNV is not always necessary for cell malignancy. When there is no internal confidential reference cell, the CNV-based method becomes unreliable. For example, when the proportion of tumor and normal cells in a sample is seriously imbalanced, the malignancy scores usually follow a unimodal distribution.

To improve the identification of malignant cells, we designed an XGBoost classifier by integrating the scRNA-seq dataset and the TCGA dataset. It is a supplement to the CNV-based method. The pipeline includes reference dataset annotation based on the bimodal distribution of malignancy score, feature selection based on pan-cancer malignant features extracted from the TCGA dataset, training an XGBoost classifier, and performance evaluation (Section 2.2). We collected a total of 663 857 cells in 175 samples (Supplementary Table S4) and constructed a high-quality single-cell reference dataset with 466 468 cells and 2756 genes. To ensure the reliability of the classifier, it is vital to balance the proportion of normal and malignant cells. Moreover, multiple cancer types were required in the reference and query sets for better pan-cancer generalization ability. Consequently, we divided the reference and query sets by sample sources instead of random sampling (Supplementary Table S4).

**Figure 4.**
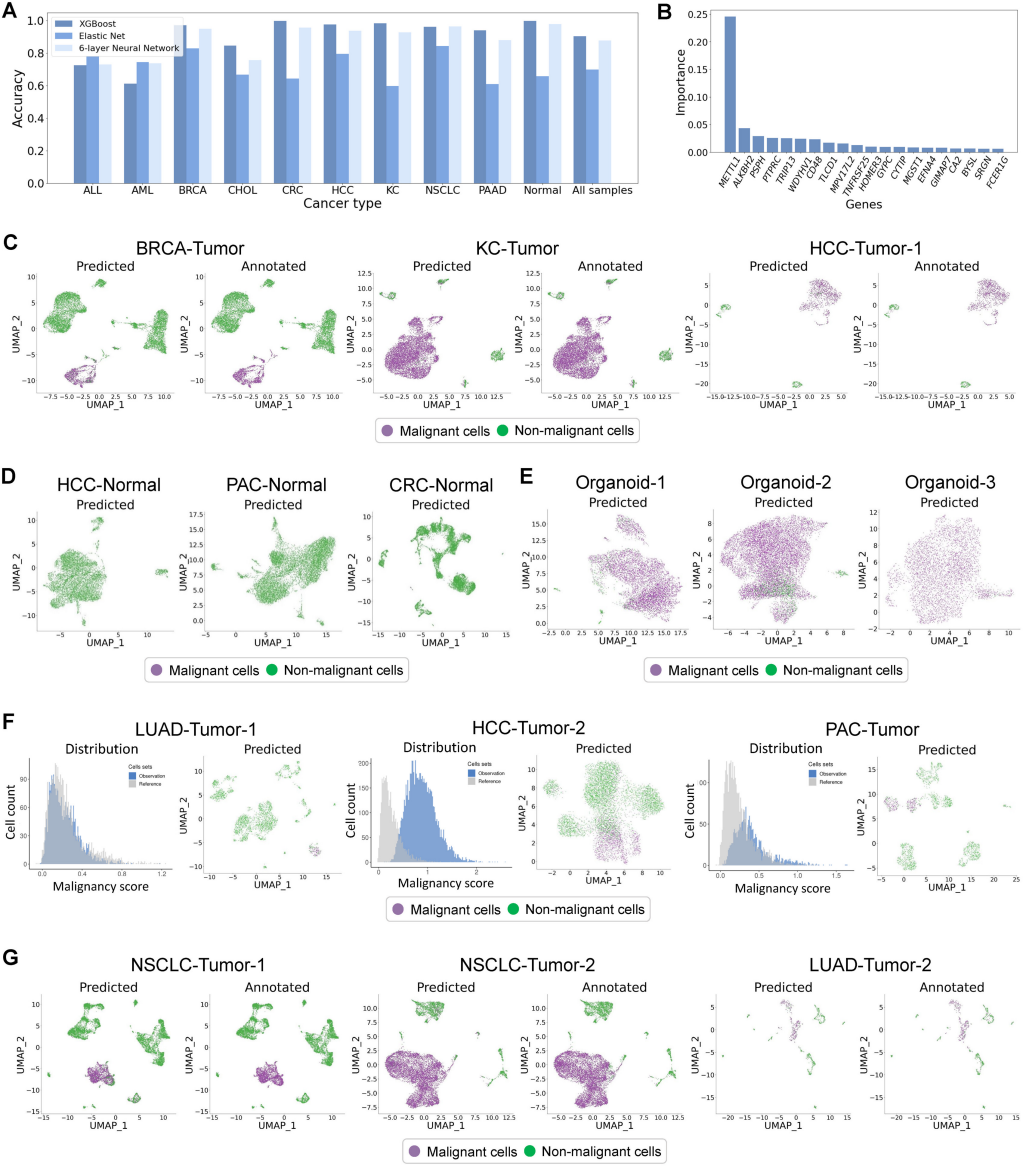
The results of malignant cell identification by scCancer2. (A) Performance comparison of different machine learning methods across multiple cancer types on malignant cell identification task. (B) Important genes for cell malignancy. The histogram shows the feature importance ranking calculated by the XGBoost model during the training process. (C) Case 1: Test scCancer2 on tumor samples with bimodal distribution of malignancy score. For each subfigure (cancer type), the left panel shows the annotation results of scCancer2 (XGBoost), while the right panel shows the annotation results of scCancer (CNV-based method). (D) Case 2: Test scCancer2 on normal samples. The UMAP-plot shows the annotation results of scCancer2 on three samples without malignant cells. (E) Case 3: Test scCancer2 on organoid samples. The UMAP-plot shows the annotation results of scCancer2 on three *in vitro* cholangiocarcinoma organoid samples. (F) Case 4: Test scCancer2 on tumor samples with unimodal distribution of malignancy score. For each subfigure, the left panel shows the distribution of malignancy score, while the right panel shows the annotation results of scCancer2. (G) Case 5: Test scCancer2 on new cancer types. The subfigures compare the prediction results and ground truth on three lung cancer samples. See Supplementary Table S4 for the correspondence between the original name of the datasets and their naming in figures.

Then, ElasticNet, XGBoost, and Neural Network were applied respectively to identify malignant cells in scRNA-seq data. The results show that XGBoost achieved better performance compared with ElasticNet and Neural Network (Fig. 4A and Supplementary Fig. S7). Moreover, the XGBoost significantly improves computational efficiency and reduces memory usage compared with the CNV-based method, especially when processing integrated large-scale datasets (Peng *et al.* 2019, Zhang *et al.* 2020). By observing the significant genes selected by the XGBoost during the training process, we judged the consistency between biological prior knowledge and the data-driven method. In particular, we noted that cancer-related genes including *METTL1*, *ALKBH2*, *PSPH*, *PTPRC*, and *TRIP13* contributed the most to the identification, which indicated the great biological interpretability of our model (Fig. 4B).

To evaluate the robustness of scCancer2, we applied it to some test samples in four distinct scenarios. Firstly, when the malignancy scores of tumor samples show a significant bimodal distribution, the annotation results of scCancer2 (XGBoost) on malignant cells are highly consistent with the annotation results of scCancer (Guo *et al.* 2021) (CNV-based method) (Fig. 4C and Supplementary Fig. S8). Secondly, when the proportion of malignant cells and normal cells is imbalanced, such as the normal samples and the tumor organoid samples, scCancer2 can accurately identify normal cells (Fig. 4D and Supplementary Fig. S9) and malignant cells (Fig. 4E and Supplementary Fig. S9), while the CNV-based method is faced with limitations (Supplementary Fig. S10). For the cancer organoid samples, the annotations can provide valuable information to evaluate the quality of *in vitro* culture. Furthermore, the CNV-based method is unreliable for a minority of solid tumor samples. When the malignancy score of the sample follows a unimodal distribution, scCancer2 can also identify malignant cells (Fig. 4F). We found that the identified malignant clusters were highly consistent with epithelial cells predicted by scCancer (Guo *et al.* 2021) in solid tumor samples (Supplementary Fig. S11). Lastly, scCancer2 can effectively annotate the malignant cells in cancer types that do not exist in the training set. In the experiment, we moved the lung cancer (LC) samples from the training set into the validation set. Then, we trained a new classifier and directly predicted the malignant cells in LC samples. A similar experiment was conducted on samples of gastric cancer. We found that scCancer2 still performed well on these samples (Fig. 4G and Supplementary Fig. S12).

In summary, the results indicate that scCancer2 effectively extracted the transcriptome characteristics of malignant cells in TME, obtaining great generalization ability across cancer types. It is a great supplement and improvement to the CNV-based method.

### 3.5 scCancer2 analyzes TME from a spatial perspective

Sequencing-based spatial transcriptomics has helped researchers to achieve impressive results in exploring the spatial heterogeneity of TME (Elhanani *et al.* 2023). scCancer2 provides a highly automated module for tumor ST analysis, which mainly consists of two parts. The first part focuses on quality control (QC), statistical analyses, and basic downstream analyses. The second part analyzes TME from three different angles.

We first utilized spatial information to perform morphology QC to filter small isolated tissue areas, which often do not contribute to the analysis. Then, we visualized unique molecular identifier (UMI) counts and the detected gene numbers to reflect the characteristics of the tissue, and filtered the spots with extremely low UMI counts or gene numbers. We provided statistical results of UMI counts on 60 tumor samples from nine cancer types (Supplementary Fig. S13 and Supplementary Table S6). Furthermore, we performed statistical analysis of gene expression proportion, especially for mitochondrial genes and ribosomal genes (Supplementary Fig. S14). After QC, we performed basic downstream analyses including dimension reduction, clustering, differential expression analysis based on Seurat (Butler *et al.* 2018), gene expression program analysis based on non-negative matrix factorization (Lee and Seung 1999), cell type and cell malignancy scoring based on marker genes (Supplementary Fig. S15).

**Figure 5.**
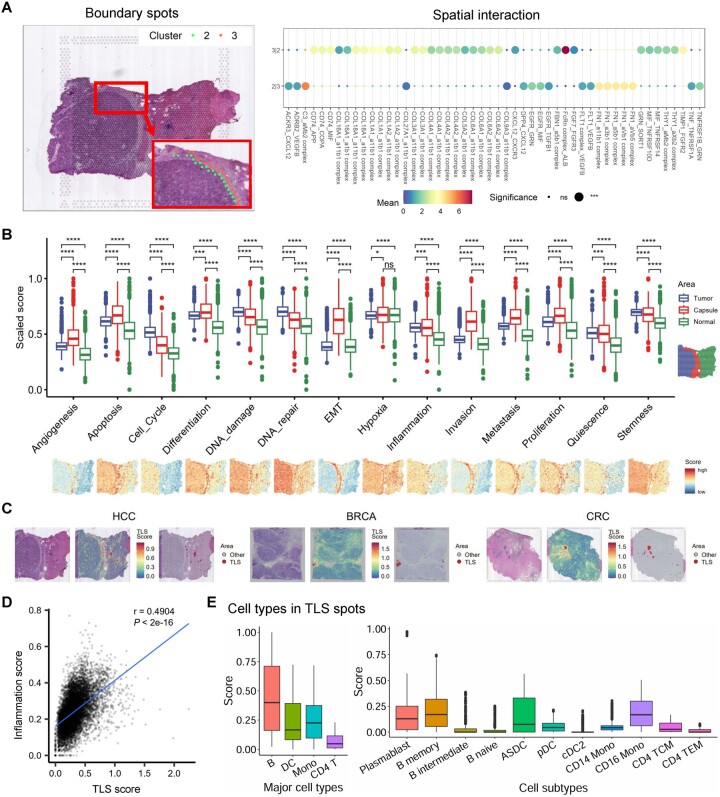
The results of TME spatial analysis by scCancer2 on tumor samples. (A) Boundary spots of neighboring clusters (left panel) and the ligand–receptor interaction strength between two clusters (right panel) in HCC-1L. (B) Boxplot showing the scores of 14 phenotypic characteristics in tumor regions, capsules, and normal regions in HCC-1L. The scores of each characteristic were scaled between 0 and 1. The *P*-value between the two groups is calculated through the Wilcoxon test. *P < .05, **P < .01, ***P < .001, and ****P < .0001. EMT, epithelial-mesenchymal transition. (C) Tertiary lymphoid structures identified by scCancer2 and their corresponding H&E images. HCC: “HCC-1L” in Wu *et al.* (2021a); BRCA: “V1_Breast_Cancer_Block_A_Section_2” in 10x Genomics; CRC: “ST-colon3” in Wu *et al.* (2022b). (D) Correlation analysis between spatial structure (TLSs) and spatial characteristic (inflammation) on samples across various cancer types in Supplementary Table S6. (E) Major cell lineages and cell subtypes enriched in TLSs. The boxplot contained all major and minor cell types which predicted to be significantly positive in the TLS spots. The cell type labels originate from Hao *et al.* (2021).

TME spatial analysis module includes interaction identification, heterogeneity characterization, and spatial structure detection. The interaction of different regions is crucial to understanding tumor behaviors, such as growth, progression, drug response, and therapeutic effect. For instance, analyzing the tumor boundary containing malignant and non-malignant spots contributed to identifying potential therapeutic targets (Xun *et al.* 2023). Therefore, scCancer2 identified the boundary spots between clusters automatically and defined the ligand–receptor (L–R) interaction strength between two clusters as the mean of the average expression of the L–R pairs from the CellPhoneDB dataset (Efremova *et al.* 2020). As seen in Fig. 5A, scCancer2 marked the boundary between the fibrous capsule and the tumor. The results of L–R interaction indicate that the extracellular matrix receptor interaction (collagen-related L–R pairs) was strong at the boundary. Besides, several disease-related L–R pairs such as CD74-APP/COPA/MIF and CXCL12-ACKR3/DPP4 were also found at the boundary (García-Cuesta *et al.* 2019, Chen *et al.* 2021, Wu *et al.* 2021c).

As for spatial heterogeneity, in addition to the scoring of cell type and malignancy mentioned above (Supplementary Fig. S15), we considered 14 phenotypes from CancerSEA (Yuan *et al.* 2019) to further explore tumor heterogeneity and the characteristics of TME. Previous studies have demonstrated the association between TME and these phenotypes, such as angiogenesis, hypoxia, and inflammation (De Palma *et al.* 2017, Jin and Jin 2020, Singleton *et al.* 2021, Denk and Greten 2022). Take HCC-1L as an example, we found that there were significant differences in phenotypic characteristics between tumor regions, capsules, and normal regions (Fig. 5B).

Spatial structures in TME are crucial to the growth and prognosis of tumors, such as tertiary lymphoid structures (TLSs) and capsules (Dieu-Nosjean *et al.* 2014, Zhu *et al.* 2019). We developed a method to automatically identify spatial structures (Section 2.3) and applied it to recognize TLSs and capsules. It achieved good performance on various cancer types, including HCC, BRCA, CRC, etc. (Fig. 5C and Supplementary Fig. S16). Furthermore, we also found that there was a significant correlation between spatial structures and transcriptome characteristics. The detected distribution of TLSs was correlated with the distribution of inflammatory features in 60 tumor samples across nine cancer types (Fig. 5D).

Finally, we extended the module of TME cell subtype annotation to the spatial level by quantitatively evaluating the cellular composition of TLSs. We detected TLSs from tumor samples (BRCA, CRC, and HCC) (Wu *et al.* 2021a, b, 2022b) and transferred the annotations of cell subtypes from the reference dataset (Hao *et al.* 2021) to all the detected TLS spots (Section 2.3). As shown in the first panel of Fig. 5E, we detected four highly enriched immune cell types in the spots of TLSs, including B cells, dendritic cells, monocytes, and T cells, which is consistent with previous studies (Sautès-Fridman *et al.* 2019, Kang *et al.* 2021). At the cell subtype level, as shown in the second panel of Fig. 5E, plasmablasts, memory B cells, and dendritic cells are the most abundant in TLSs. Common subtypes of CD4 T cells (TCM and TEM) can also be detected.

In summary, we implemented a new module to analyze TME based on spatial transcriptomics data. This module is highly systematic and automated. It enables us to explore the spatial characteristics of TME from multiple perspectives comprehensively.

## 4 Discussion

By leveraging machine learning and integrating multiple analysis modules, we provided a valuable tool scCancer2 for researchers to gain a more comprehensive understanding of the TME at the single-cell level. Our analysis consists of three different modules. Firstly, we trained a series of machine-learning models on scRNA-seq data for cell subtype annotation and quantitatively evaluated the similarity of labels originating from different datasets. Secondly, we constructed a reference dataset based on bulk RNA-seq and scRNA-seq data and identified the malignant cells with the XGBoost classifier. Thirdly, we integrated a module of ST analysis for a multi-dimensional view of TME.

For the cell subtype annotation module, we tested classic machine learning methods (e.g. SVM and XGBoost) and specialized algorithms for scRNA-seq on massive data. The evaluation of classification algorithms requires consideration of multiple factors: classification performance, computational efficiency, model complexity, and biological interpretability. In terms of accuracy within a single dataset, scCancer2 is close to SVM in scPred (Alquicira-Hernandez *et al.* 2019). However, scCancer2 can generate lightweight models and rapidly assign multiple sets of labels to new input data. Meanwhile, we found that XGBoost achieved better performance than other algorithms on classification tasks (Supplementary Fig. S17), but its generalization ability was worse than scCancer2 on cross-dataset annotation, making it hard to discover the relationship of labels originating from different datasets (Supplementary Figs S18 and S19).

The results of cross-dataset annotation indicate the generalization ability of scCancer2 on immune cells and endothelial cells. Cell subtypes with similar functions showed high similarity in the heatmap (Supplementary Figs S2–S5). The results in fibroblasts are relatively ambiguous (Supplementary Fig. S6) due to the heterogeneity of fibroblasts (Kanzaki and Pietras 2020, Lavie *et al.* 2022). scCancer2 provides a convenient access for users to not only extract specific cell subtypes from the dataset but also index similar subtypes and relevant references. We hope to propose reliable indicators for quantitatively evaluating the cross-dataset performance of algorithms based on similarity maps.

For the malignant cell identification module, machine learning-based methods in scCancer2 can be a great supplement to the CNV-based method. Firstly, scCancer2 extracts the transcriptome characteristics of malignant cells and performs well when the CNV-based method fails to work. Secondly, when directly processing large-scale published datasets instead of individual samples, the time cost and memory burden of the CNV-based method are high, while the trained model in scCancer2 can rapidly identify malignant cells in large-scale datasets.

For the ST analysis module, exploring tumor heterogeneity from a spatial perspective is very significant. As we have already analyzed the composition of TLSs at the cell subtype level, we hope to benchmark more deconvolution and mapping strategies for the integration of scRNA-seq and spatial transcriptomics data (Longo *et al.* 2021). Besides, transcriptome characteristics extracted from bulk RNA-seq and scRNA-seq data such as malignancy features can also be utilized for spatial heterogeneity analysis.

In the future, we will pay more attention to the scalability of scCancer2. In recent studies, there are several newly discovered or rare cell subtypes (Hanada *et al.* 2022, Nalio Ramos *et al.* 2022). Detecting them in the existing samples is an important issue. We can treat these cell subtypes as positively labeled queries and solve this problem with novelty detection methods such as one-class models (Perera *et al.* 2021). Moreover, adding new subtypes to existing label networks rapidly (without cross-dataset annotation) is challenging. Co-modeling the expression matrix and the label similarity matrix as a label network by graph models cannot only visualize the relationship more clearly, but also provide access for new nodes by graph embedding and link prediction.

## Supplementary Material

btae028_Supplementary_Data
