# Supplementary material for: scCancer2: data-driven in-depth annotations of the tumor microenvironment at single-level resolution
Source: Bioinformatics. 2024 Jan 18;40(2):btae028. doi: 10.1093/bioinformatics/btae028 (PMC10868330; doi:10.1093/bioinformatics/btae028)
Supplement: btae028_Supplementary_Data [file btae028_supplementary_data.pdf]

Supplementary Information

Supplemental Figures

Similarity map of major cell subtypes on query datasets

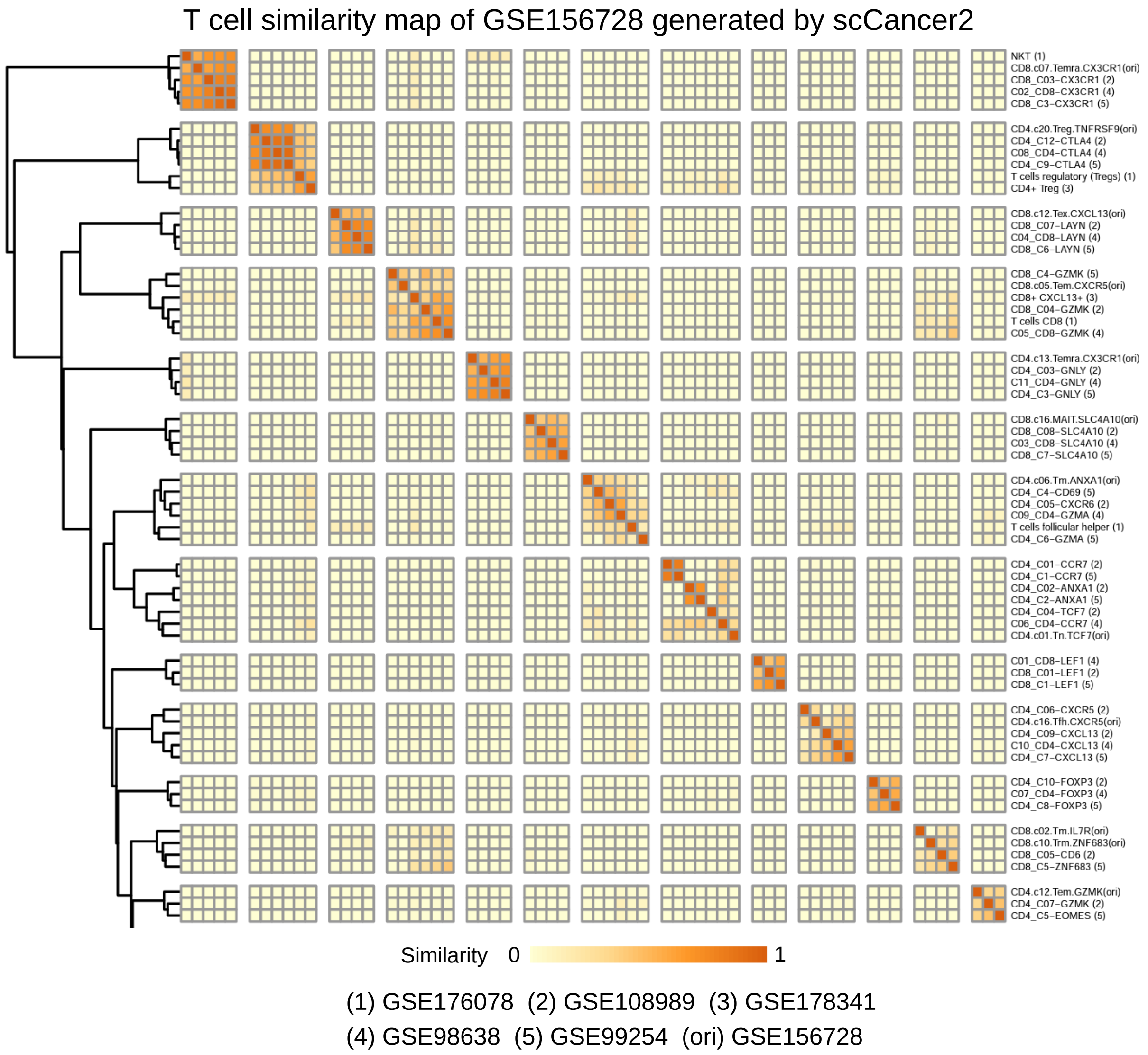

Fig. S1. Similarity map of T cell subtypes on GSE156728. The original annotation of T cells were used for comparison.

Integrated similarity map of major cell subtypes on multiple training datasets generated by scCancer2

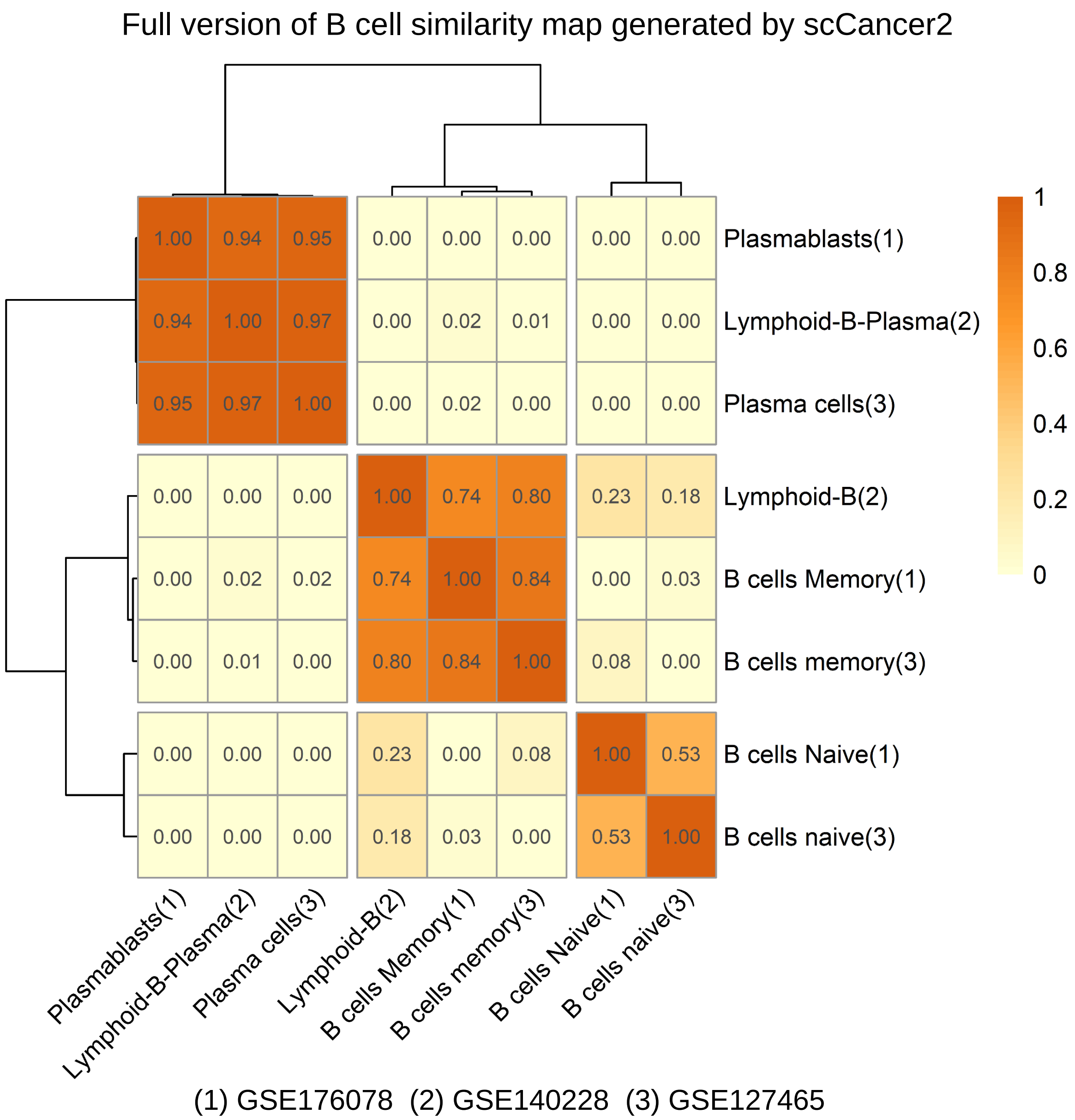

Fig. S2. Similarity map of B cell subtypes generated from cross-dataset annotation (3 training sets).

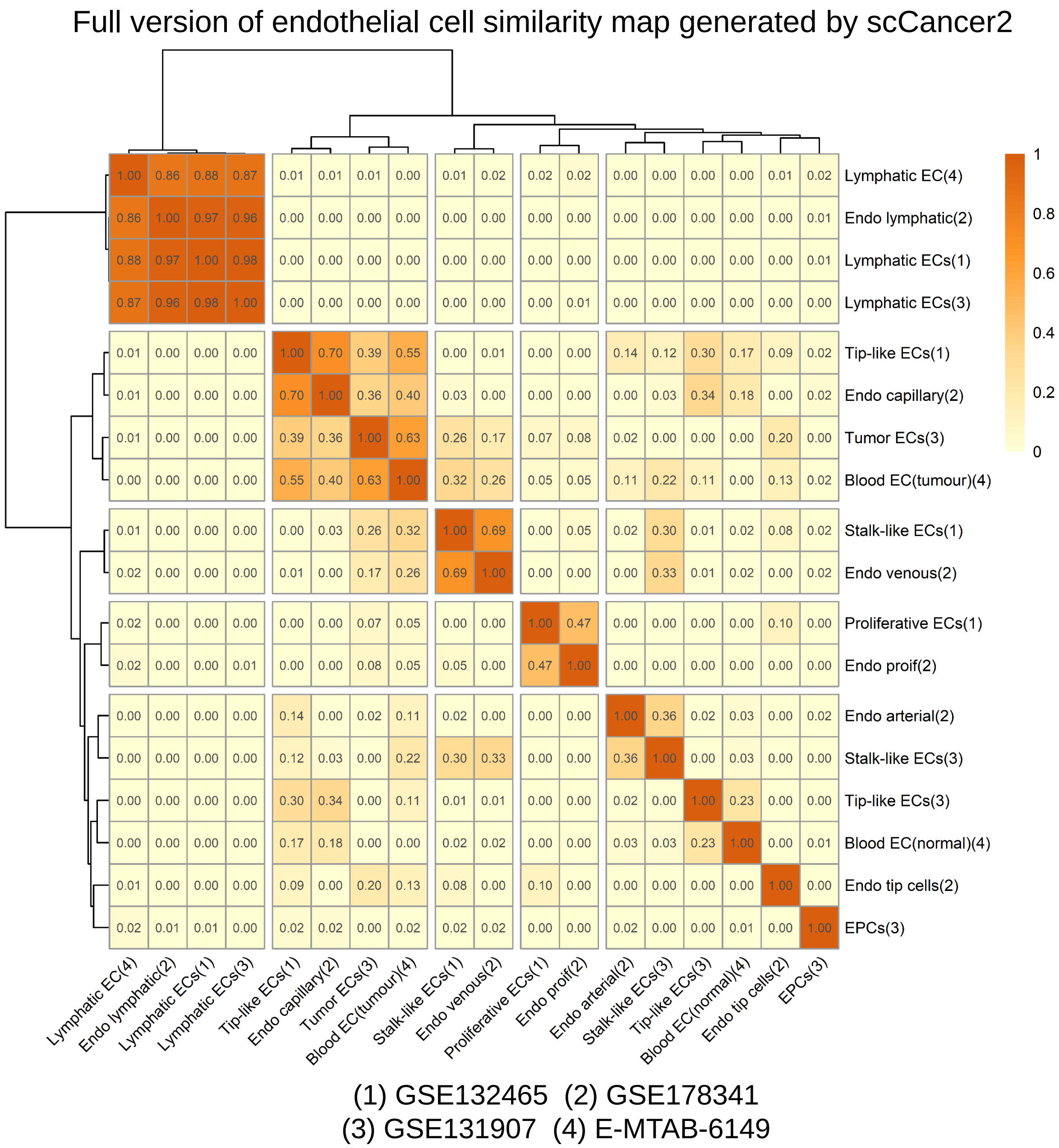

Fig. S3. Similarity map of endothelial subtypes generated from cross-dataset annotation (4 training sets).

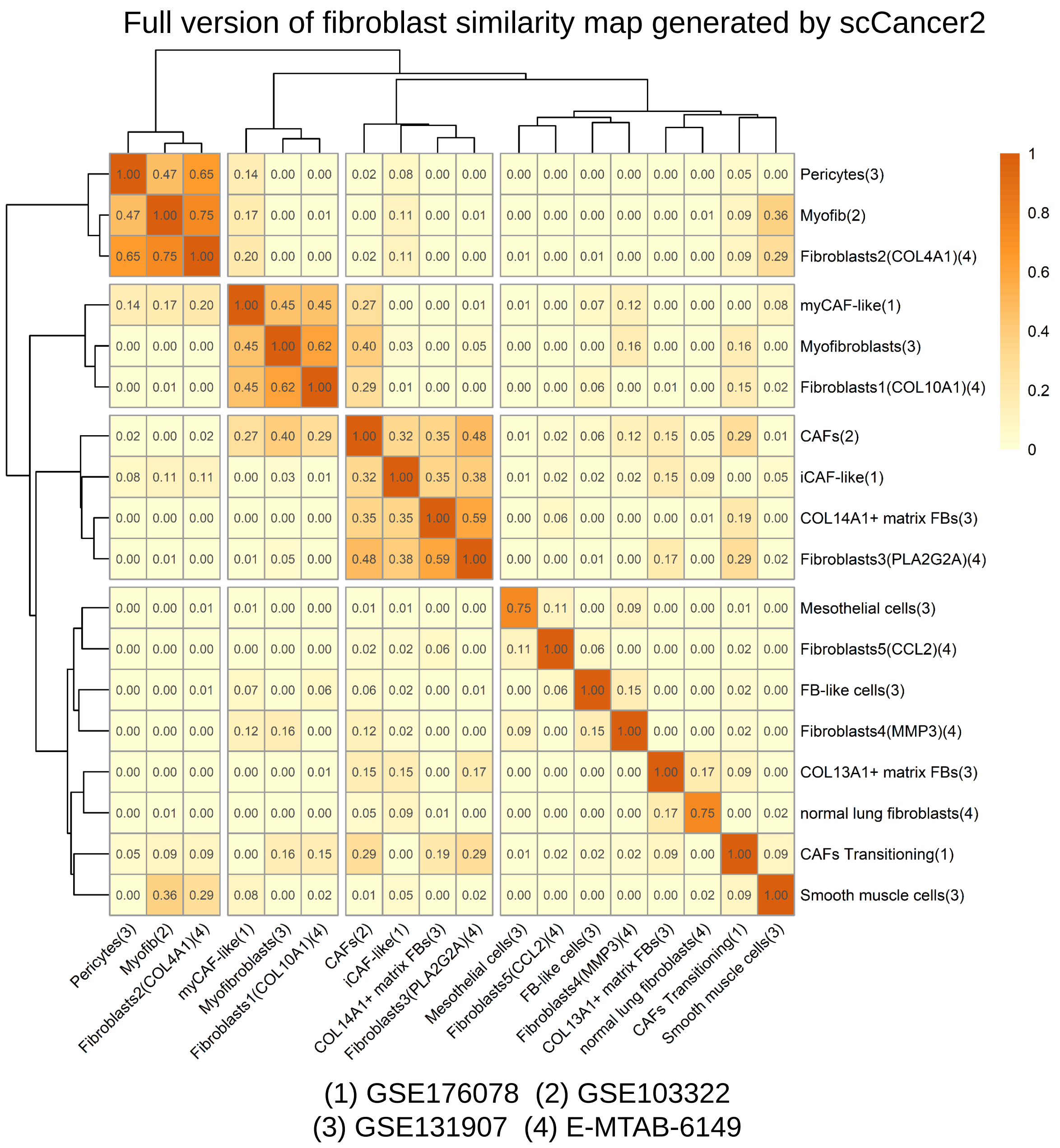

Fig. S4. Similarity map of fibroblast subtypes generated from cross-dataset annotation (4 training sets).

Full version of myeloid cell similarity map generated by scCancer2

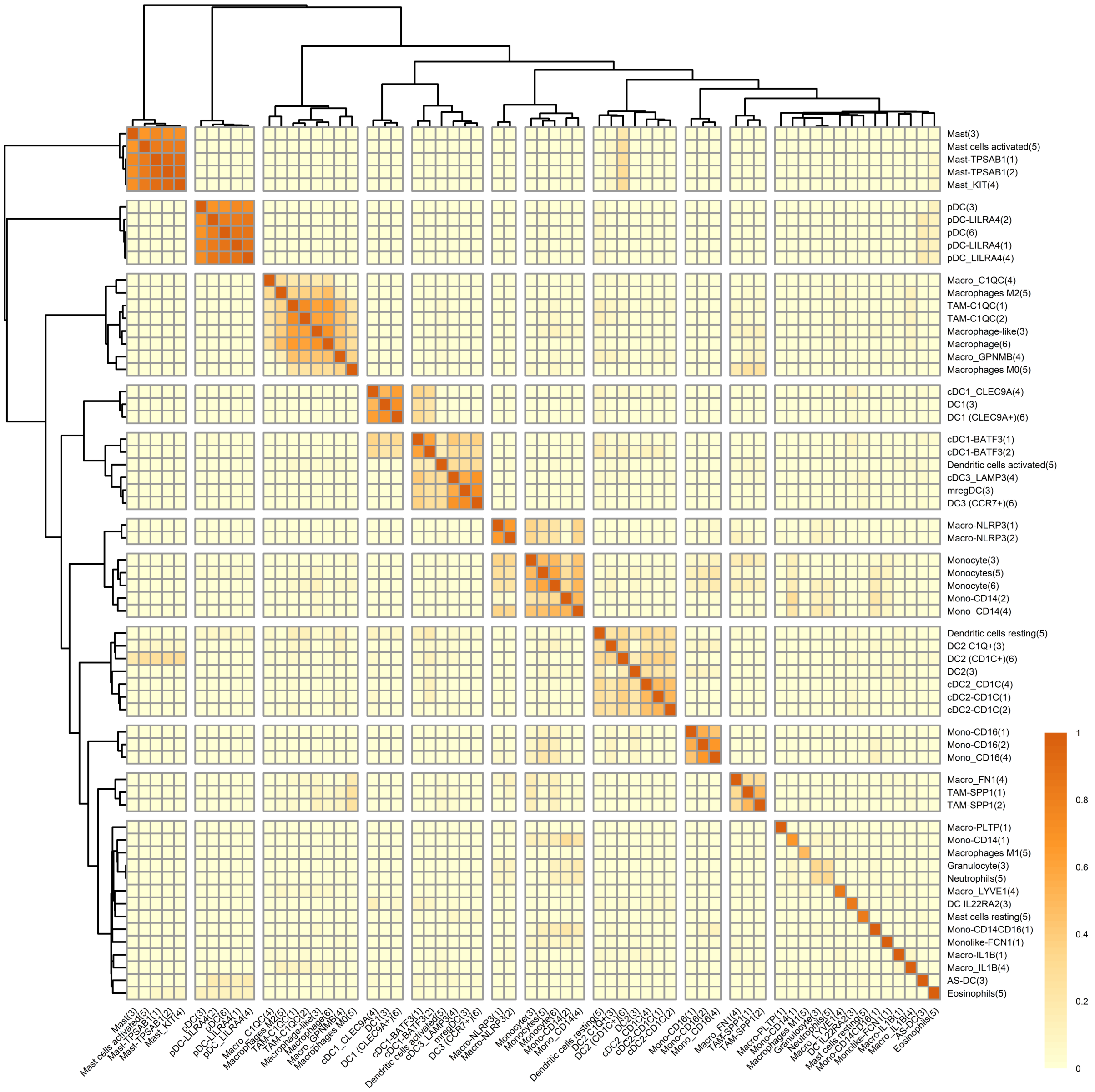

(1) GSE146771-10X (2) GSE146771-SmartSeq2 (3) GSE178341
(4) GSE154763 (5) GSE127465 (6) GSE150430

Fig. S5. Similarity map of myeloid subtypes generated from cross-dataset annotation (6 training sets).

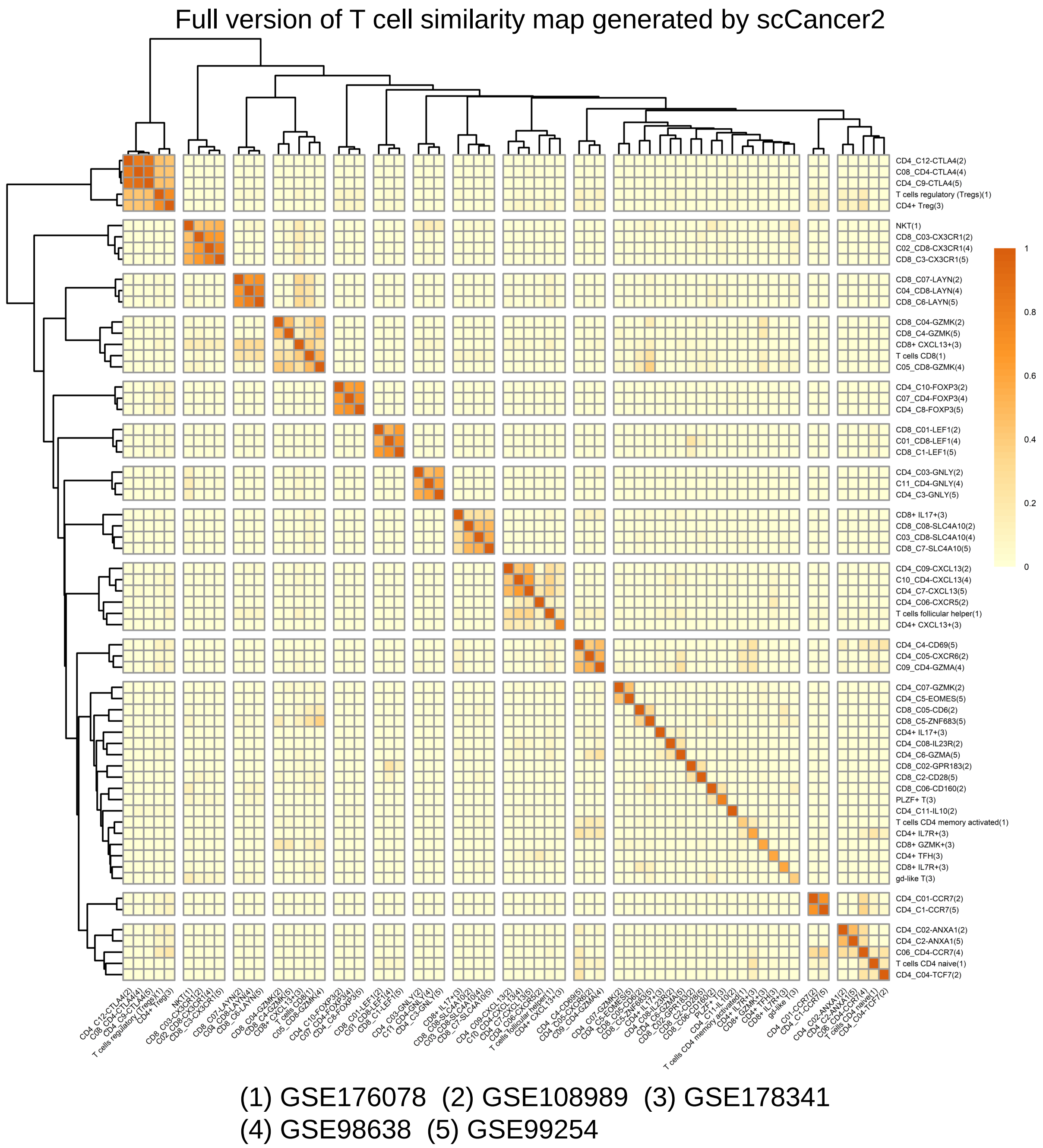

Fig. S6. Similarity map of T cell subtypes generated from cross-dataset annotation (5 training sets).

More results on malignant cell identification task

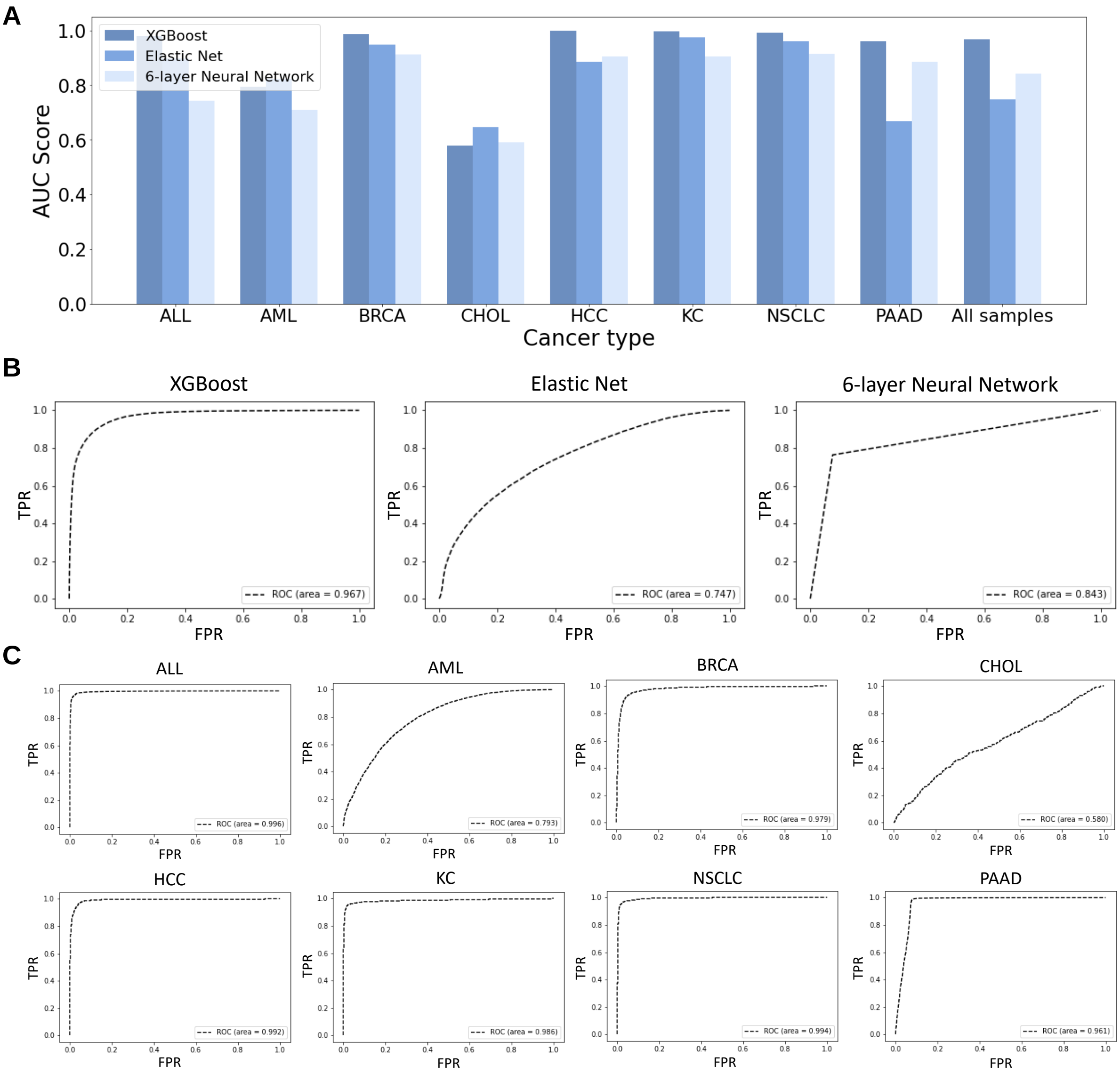

Fig. S7. Supplemental results of Fig. 4A (applying ROC curves and AUC scores as additional indicators).
(A) AUC scores of three models on multiple cancer types. **(B)** ROC curves of three models on all samples. **(C)** ROC curves of XGBoost model on multiple cancer types. * Note: Compared with **Fig. 4A**, there is no "CRC" because the CRC dataset used for performance evaluation only contains immune cells (non-malignant cells). ROC curve and AUC score can not be generated.

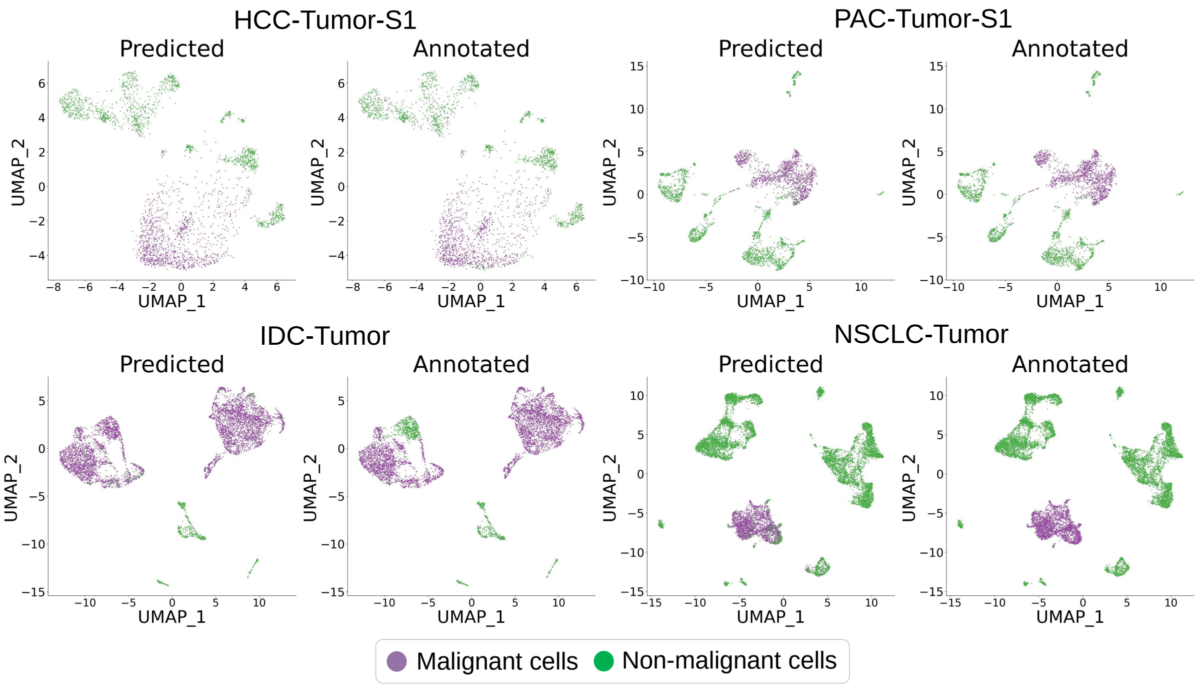

Fig. S8. Supplemental results of case 1. Tumor samples with bimodal distribution of CNV score. The annotations of scCancer2 (XGBoost model) showed high consistency with CNV-based method.

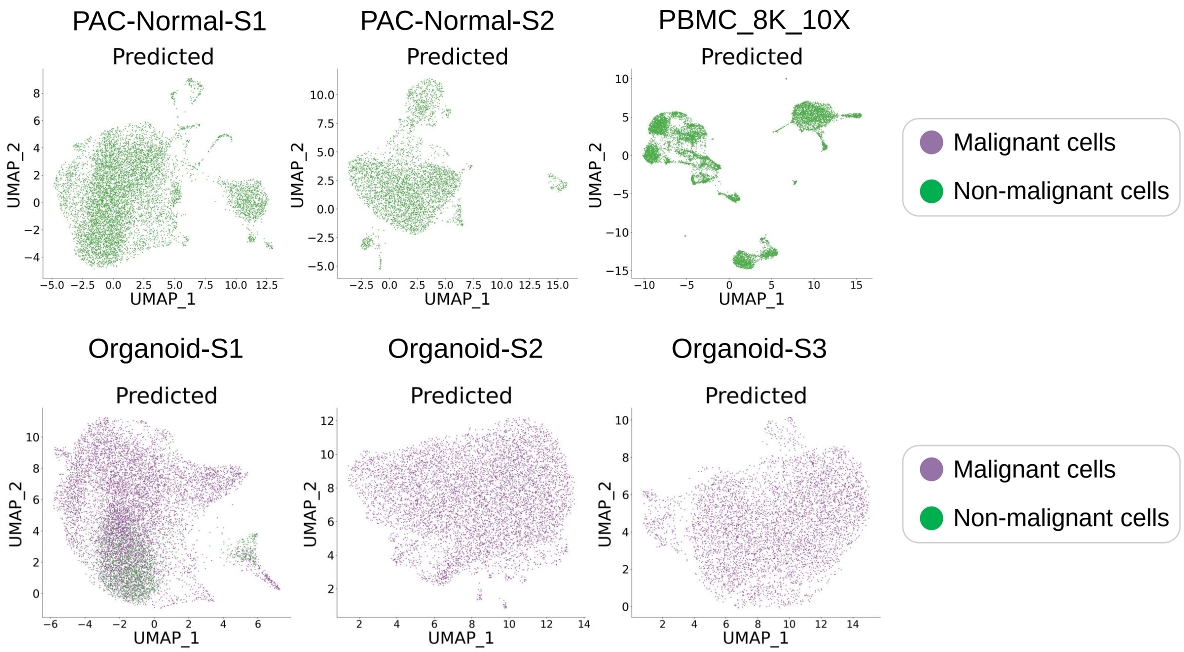

Fig. S9. Supplemental results of case 2 and case 3 (performance of scCancer2). Normal samples and organoid samples. scCancer2 (XGBoost model) can effectively identify tumor or normal cells when CNV-based method fails to work.

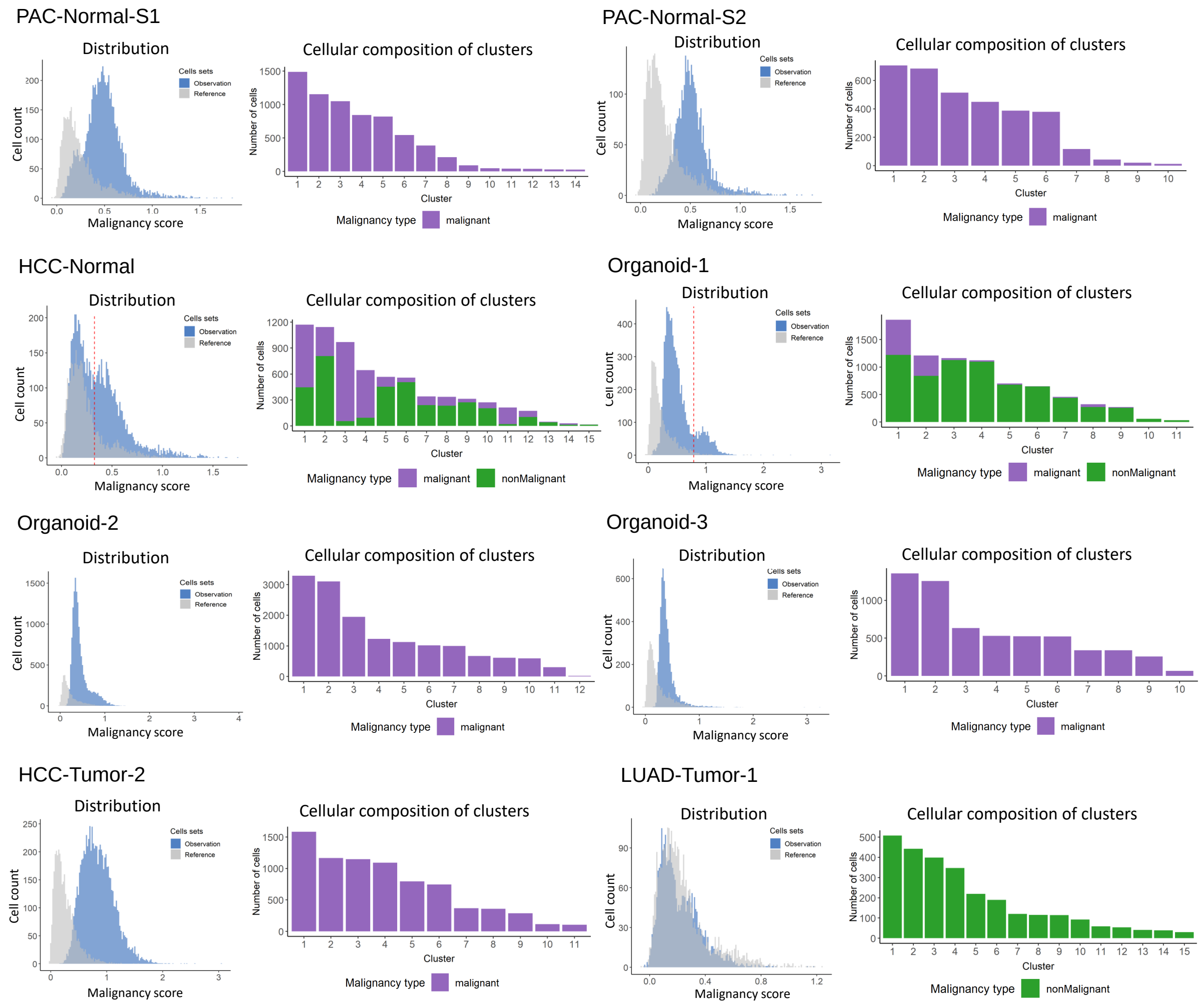

Fig. S10. Supplemental results of case 2 and case 3 (performance of CNV-based method). CNV-based method has limitations on imbalanced samples. The results were generated by old version of scCancer (cell malignancy estimation based on inferCNV).

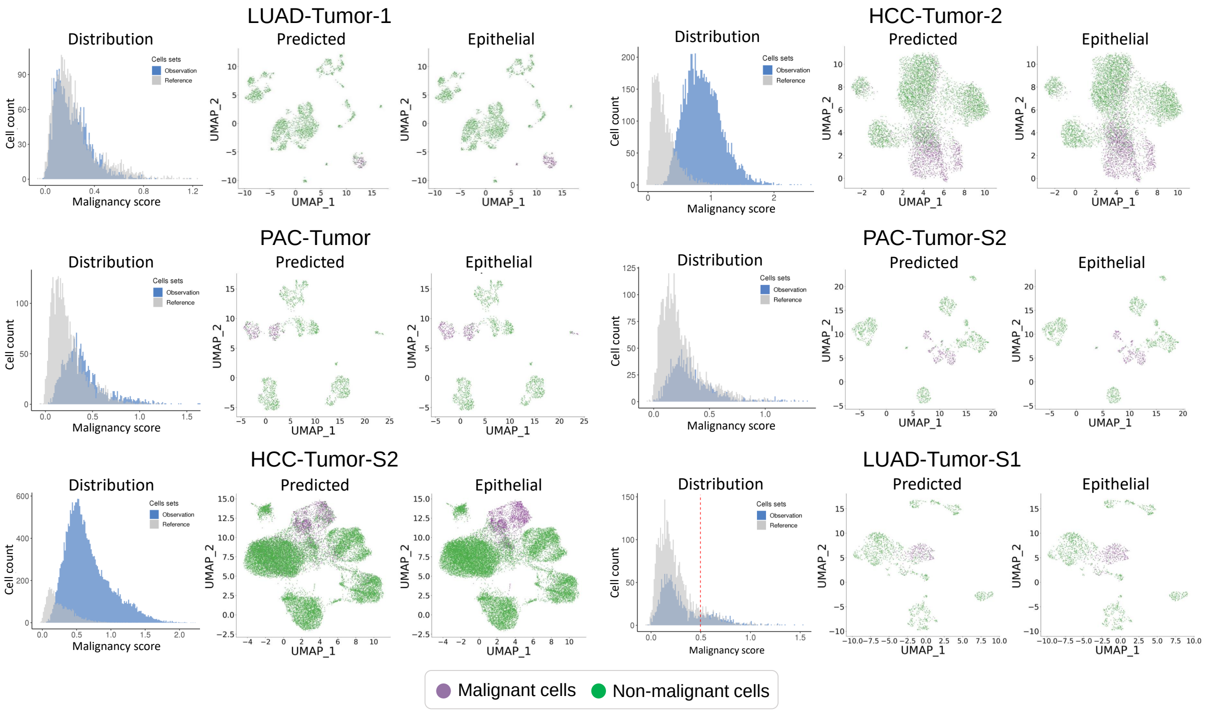

Fig. S11. Supplemental results of case 4. Tumor samples with unimodal distribution of CNV score. scCancer2 (XGBoost model) can effectively identify malignant clusters when CNV-based method fails to work. "Epithelials" was annotated by old version of scCancer.

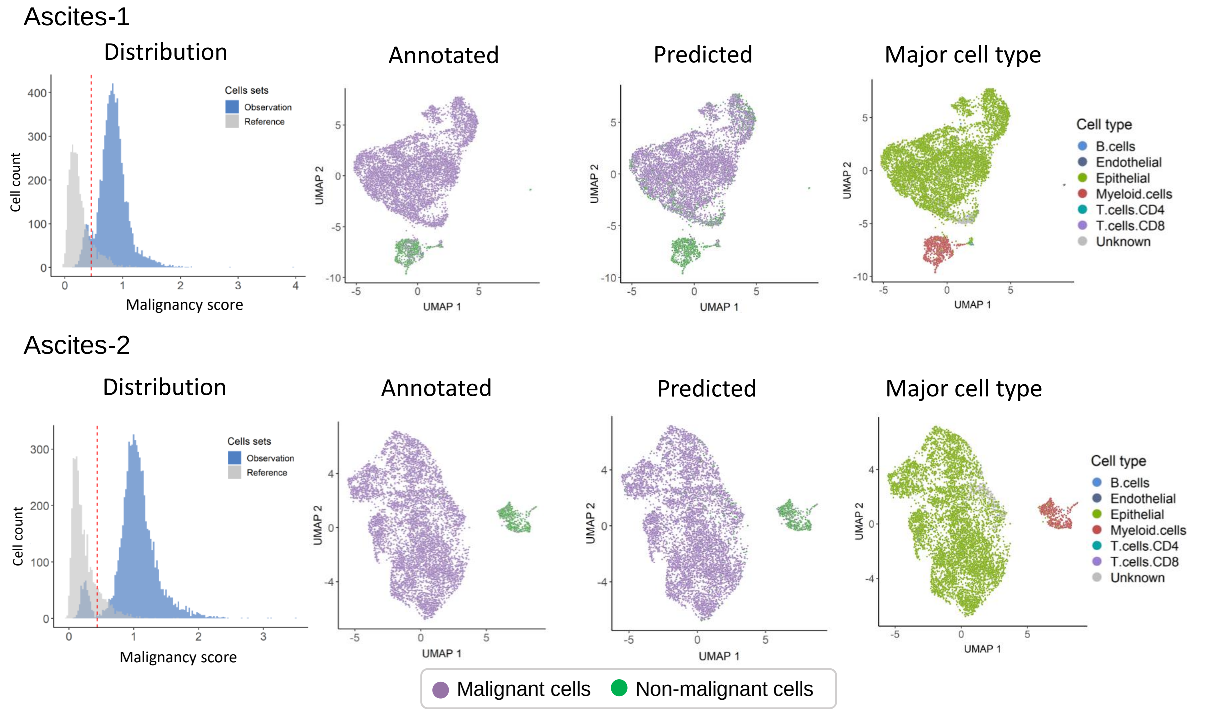

Fig. S12. Supplemental results of case 5. scCancer2 effectively annotate the malignant cells in cancer types that do not exist in the training set. The two samples were collected from ascites of gastric cancer patients. "Major cell type" was annotated by old version of scCancer.

More results on spatial transcriptome analysis

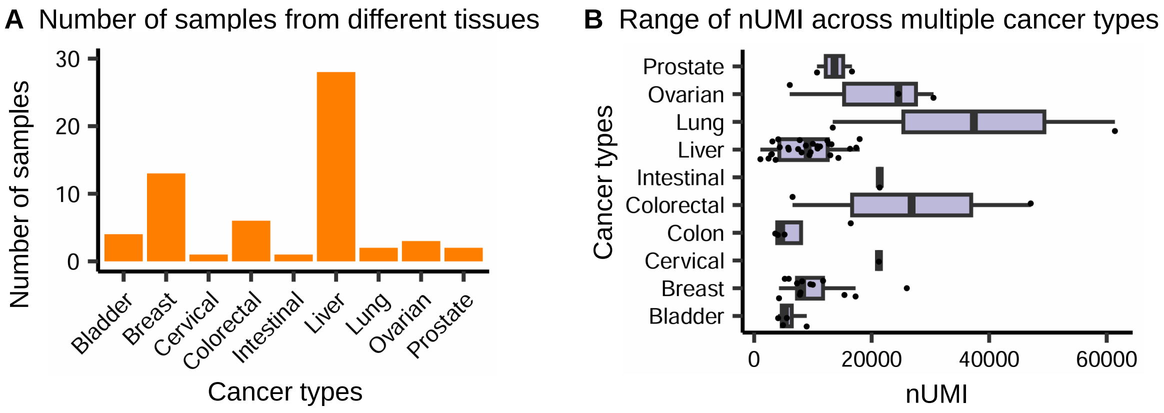

Fig. S13. Statistical results of tumor samples from different tissues.

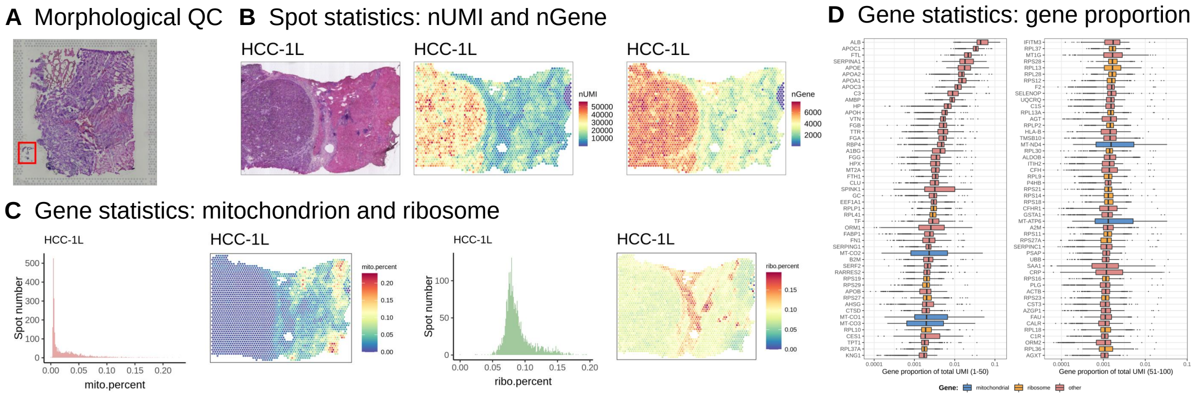

Fig. S14. Supplemental results of stStatistics module in scCancer2 on HCC-1L.

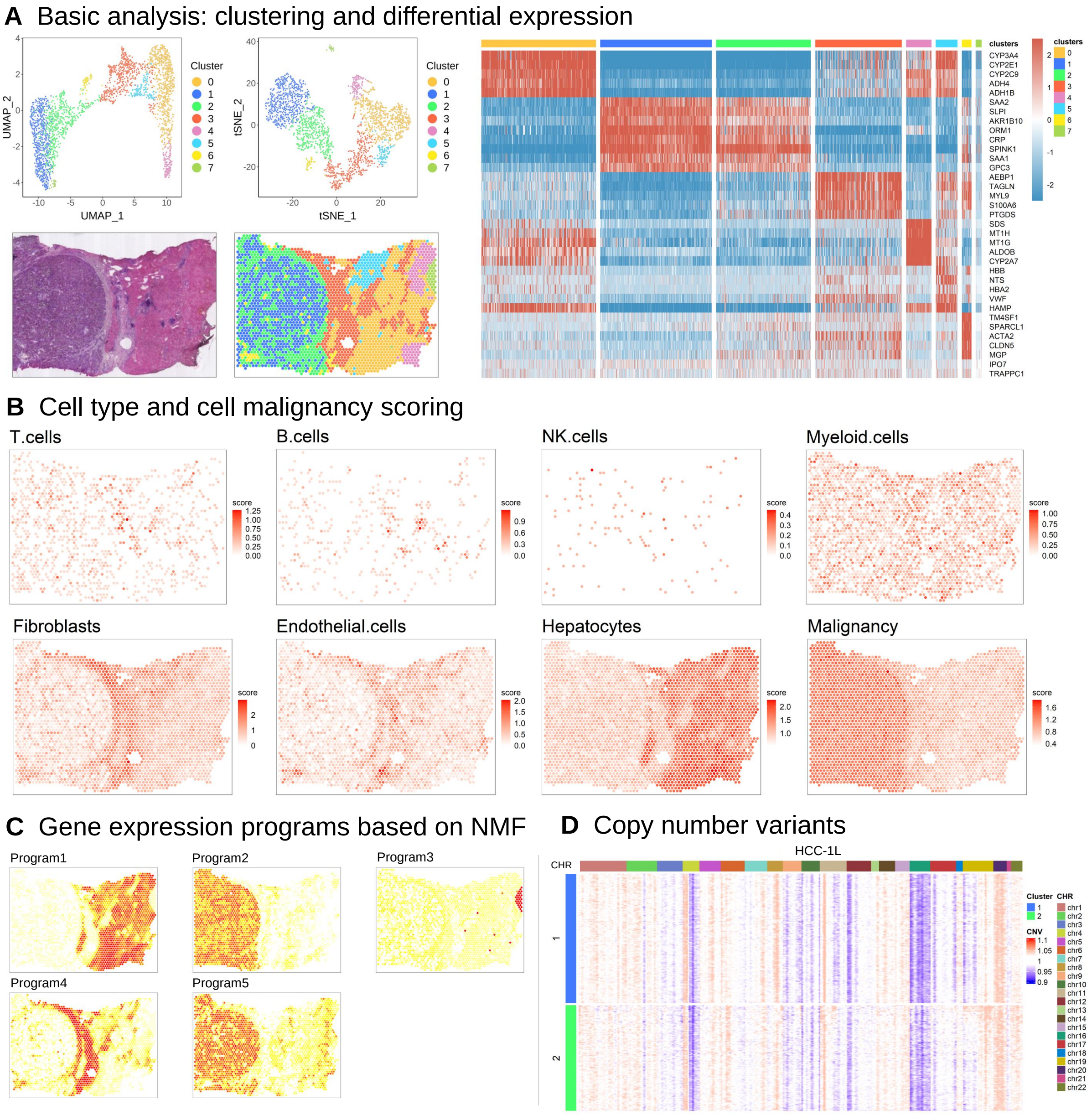

Fig. S15. Supplemental results of stAnnotation module in scCancer2 on HCC-1L.

Implementation details of Fig. S14 and Fig. S15

Quality control

We first utilized spatial information to perform morphological quality control on spots. In some samples, isolated tissue areas may appear in the section, which often does not contribute to the analysis results, and even bring errors to clustering, and differential gene analysis. Therefore, we removed these small regions through connected domain processing.

For 10X Visium data, we obtained the 6 nearest neighbors of each spot and constructed connected domains. By default, regions with connected domain area less than 3 are removed. The remaining area will be used for the following steps. Then, we visualized the detected gene numbers and nUMI in the tissue image and filtered the spots with extremely low gene numbers or nUMI. In addition to QC on spots, we also performed QC on genes. First, we filtered genes that expressed in less than 3 cells. Then we represented the expressions of mitochondrial genes and ribosomal genes, and it is suggested that researchers carefully consider the effects of these genes on the final results.

Basic downstream analysis

After quality control steps, we performed basic downstream analyses based on Seurat, including normalization, highly variable genes identification, dimension reduction (PCA/tSNE/UMAP), clustering, and differential expression analysis. Then we represented them with some redesigned visualization functions.

Cell type and cell malignancy scoring

Marker genes of common cell types from Wu et al. (2021a) were used to roughly estimate the cell distribution and cell composition in each spot. We defined the cell type (malignancy) evaluation scores as the average expression of marker genes.

Gene expression program analysis

Non-negative matrix factorization (NMF) Lee and Seung (1999) can identify gene expression programs unsupervisedly. Each column of the left matrix obtained after NMF decomposition can be regarded as the relevant gene of a program extracted, and each column of the right matrix can be regarded as the strength of each program expressed by the tissue. In scCancer2, we applied NMF decomposition to the normalized expression matrix to reveal expression programs. And the intensities of each expression program are shown in figures.

Copy number variation analysis

Copy number variation is the potential to identify malignant tumor cells, which was widely used in the research of various cancer types. Here, we integrated the algorithm of R packages inferCNV and CopyKAT Gao et al. (2021) to estimate CNVs of each spot. InferCNV was to calculate the moving average of expression values across each chromosome and then compared them with normal reference data to estimate CNVs. Considering the impact of dropout, we

used spots' neighbor information of PCA space to smooth CNV values. CopyKAT combined a Bayesian approach with hierarchical clustering to calculate genomic copy number profiles of single cells and defined clonal substructure. This method only required a gene expression matrix of UMI counts and then outputted the copy number profiles and predicted labels of cells. If users have reference data, we recommend using inferCNV because it is faster. If not, we recommend using CopyKAT to calculate CNVs automatically.

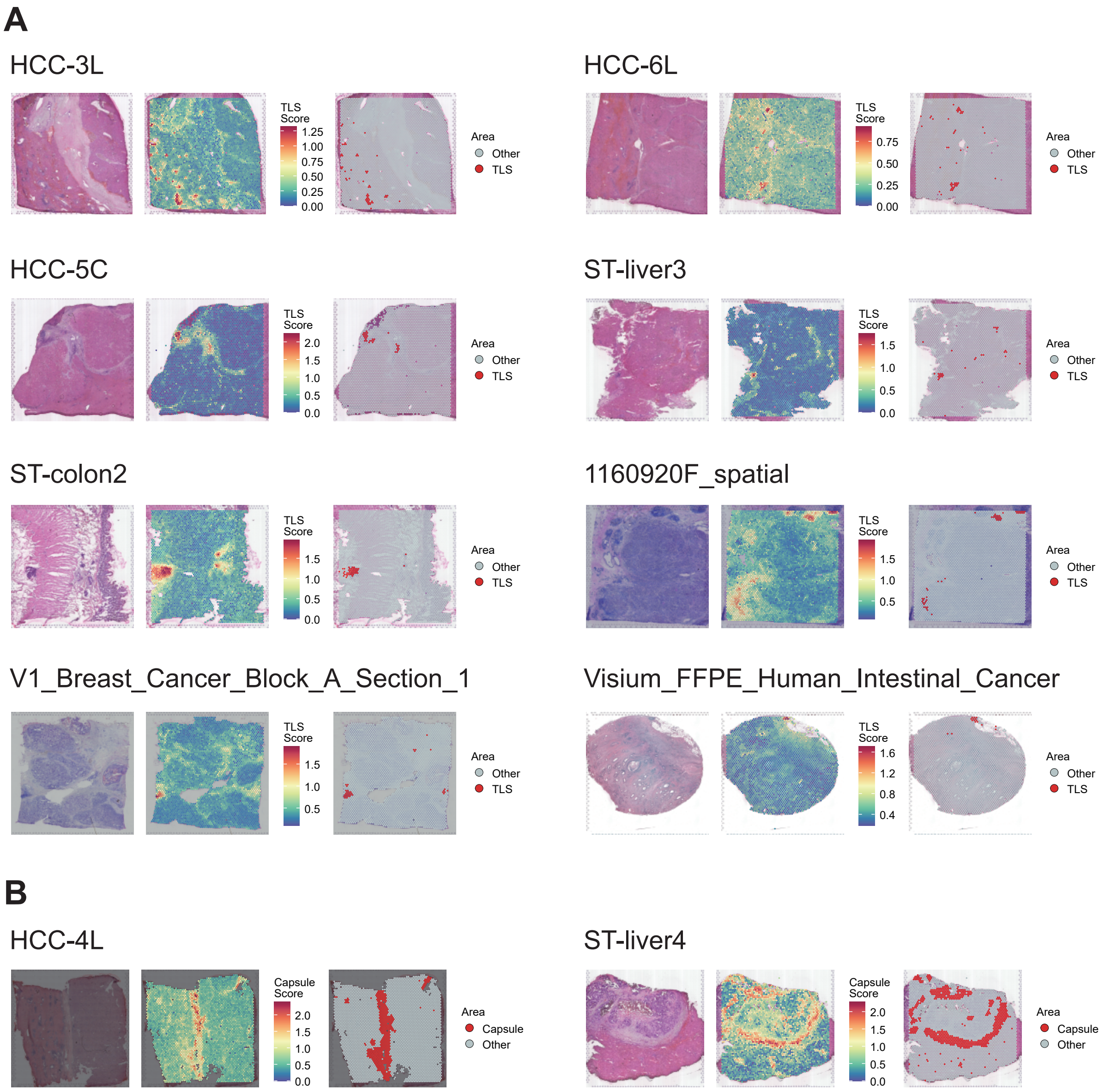

Fig. S16. Supplemental results of spatial structure detection.
(A) TLSs identified by scCancer2 and their corresponding H&E images. **(B)** Capsules identified by scCancer2 and their corresponding H&E images. * "HCC-3L", "HCC-4L", "HCC-5C", "HCC-6L": Wu et al. (2021a); "ST-liver3", "ST-liver4", "ST-colon2": Wu et al. (2022b); "1160920F_spatial": Wu et al. (2021b); "V1_Breast_Cancer_Block_A_Section_1", "Visium_FFPE_Human_Intestinal_Cancer": 10X Genomics.

850 ## Performance evaluation of XGBoost on cell subtype annotation

851 ## Performance evaluation of XGBoost on every single dataset

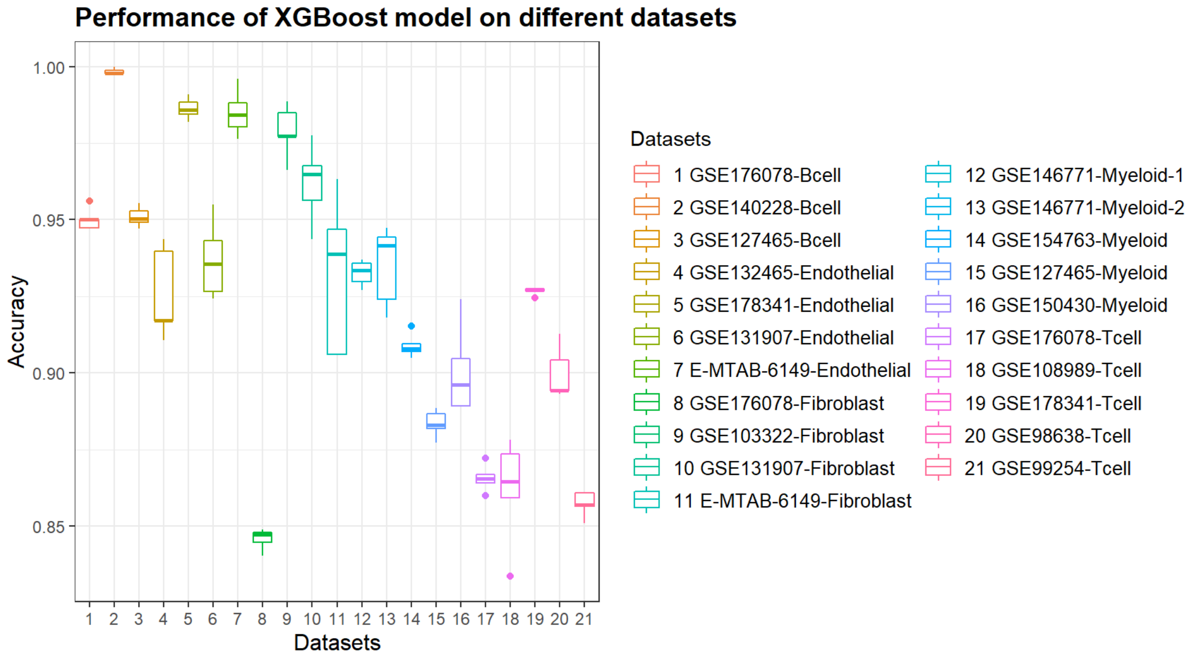

Fig. S17. Performance evaluation of XGBoost model on subtype annotation task. The results were obtained by 5-fold cross-validation.

Performance evaluation of XGBoost on cross-dataset annotation

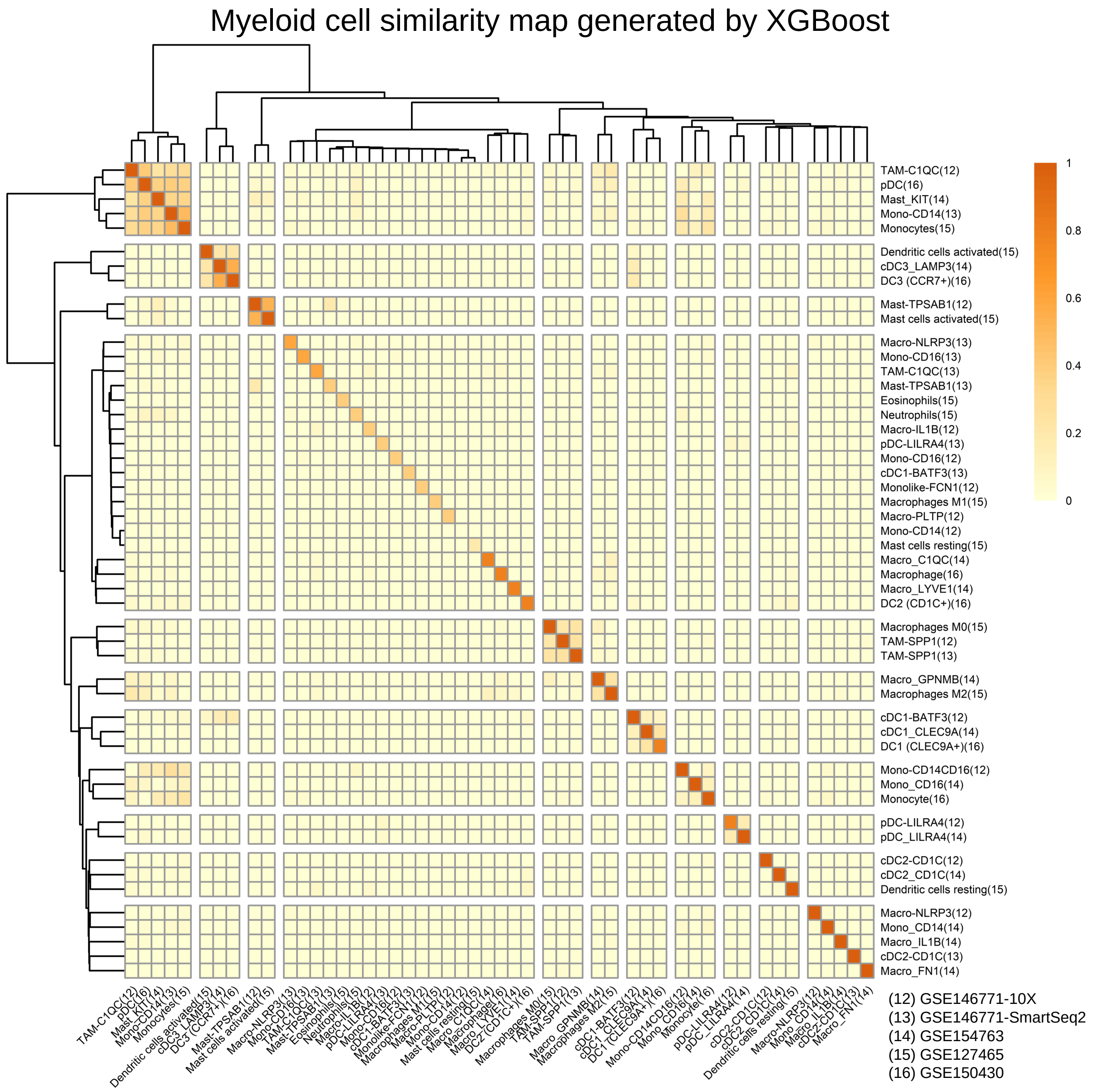

Fig. S18. Similarity map of myeloid cell subtypes generated from cross-dataset annotation (5 training sets). XGBoost model has poor cross-dataset generalization ability on myeloid cells.

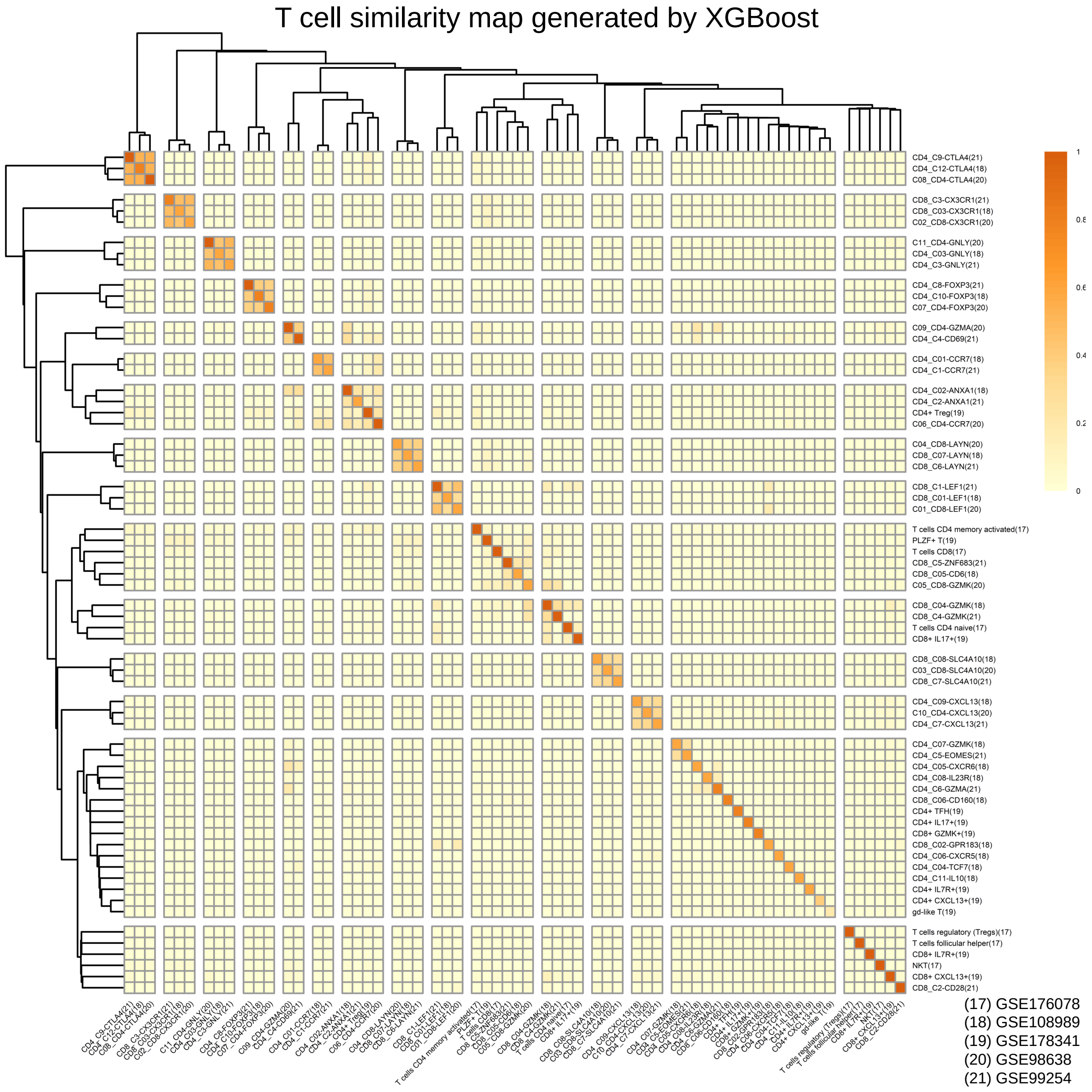

Fig. S19. Similarity map of T cell subtypes generated from cross-dataset annotation (5 training sets). XGBoost model has poor cross-dataset generalization ability on T cells.

Supplemental Tables

Table S1: Methods and packages newly implemented in scCaner2

**Steps**	**Packages and References**	**Methods**
Sample selection	Garnett (Pliner et al., 2019)	Aggregated marker scores
Feature selection	Scibet (Li et al., 2020)<br>Highly Regional Genes (Wu et al., 2022a)	Entropy-test<br>Graph-based gene selection
Cell subtype annotation	Scibet (Li et al., 2020)	Build a multinomial model<br>for each cell subtype
Malignant cell identification	xgboost (Chen and Guestrin, 2016)	Extreme gradient boosting
Cell-cell interaction in spatial<br>transcriptomics data	CellPhoneDB (Efremova et al., 2020)	Combine the expression of multi-<br>subunit ligand-receptor complexes
Copy number variation analysis	CopyKAT (Gao et al., 2021)	Calculate genomic copy number profiles of<br>single cells and defined clonal substructure

Table S2: References for cell subtype annotation in scCaner2

Major cell type	Accession	Reference[1]
B cell (3 refs)	(1) GSE176078	Breast2021, Wu et al. (2021b)
	(2) GSE140228	Liver2019, Zhang et al. (2019)
	(3) GSE127465	Lung2019, Zilionis et al. (2019)
endothelial cell (4 refs)	(1) GSE132465	Colorectal2020, Lee et al. (2020)
	(2) GSE178341	Colorectal2021, Pelka et al. (2021)
	(3) GSE131907	Lung2020, Kim et al. (2020)
	(4) E-MTAB-6149	Lung2018, Lambrechts et al. (2018)
fibroblast (4 refs)	(1) GSE176078	Breast2021, Wu et al. (2021b)
	(2) GSE103322	HeadandNeck2017, Puram et al. (2017)
	(3) GSE131907	Lung2020, Kim et al. (2020)
	(4) E-MTAB-6149	Lung2018, Lambrechts et al. (2018)
myeloid cell (6 refs)	(1) GSE146771-10X	Colorectal2020, Zhang et al. (2020)
	(2) GSE146771-SmartSeq2	Colorectal2020, Zhang et al. (2020)
	(3) GSE178341	Colorectal2021, Pelka et al. (2021)
	(4) GSE154763	Pan-cancer2021, Cheng et al. (2021)
	(5) GSE127465	Lung2019, Zilionis et al. (2019)
	(6) GSE150430	Nasopharyngeal2020, Chen et al. (2020)
T cell (5 refs)	(1) GSE176078	Breast2021, Wu et al. (2021b)
	(2) GSE108989	Colorectal2018, Zhang et al. (2018)
	(3) GSE178341	Colorectal2021, Pelka et al. (2021)
	(4) GSE98638	Liver2017, Zheng et al. (2017)
	(5) GSE99254	Lung2018, Guo et al. (2018)

[1] For each major cell lineage, the order of references corresponds to the order of annotated labels in scCancer2.

Table S3: Multi-label annotation for input TME dataset

Major cell type	Barcodes	Cell subtype	Reference
T cell	ATCG	T-subtype1.1<br>T-subtype2.1	T-ref1<br>T-ref2
	...		
	ACTG	T-subtype1.2<br>T-subtype2.2	T-ref1<br>T-ref2
myeloid cell	GCTA	M-subtype1.1<br>M-subtype2.1	M-ref1<br>M-ref2
	...		
	GTAC	M-subtype1.2<br>M-subtype2.2	M-ref1<br>M-ref2
B cell		...	
endothelial cell		...	
fibroblast		...	

Table S4: The list of scRNA-seq datasets used in malignant cell identification

Cancer type[2]	dataset[3]	No. of cells[4]	Download	Property[5]
ALL	GSE132509_11samples	37,936	TISCH2	Reference
	GSE154109_7samples	10,799	TISCH2	Query1
AML	GSE116256_21samples	38,348	TISCH2	Reference
	GSE154109_8samples	9,623	TISCH2	Query1
BRCA	GSE148673_6samples_TNBC	10,359	TISCH2	Reference
	BRCA-Tumor (GSE150660_3samples)	10,605	TISCH2	Query1
	IDC-Tumor (GSE148673_2samples_IDC)	6,196	TISCH2	Query1
CHOL	GSE138709_5samples	33,990	TISCH2	Reference
	GSE142784_2samples	10,507	TISCH2	Query1
	Organoid-1	9,331	Unpublished	Query2
	Organoid-2	16,544	Unpublished	Query2
	Organoid-3	6,982	Unpublished	Query2
	Organoid-S1	16,616	Unpublished	Query2
	Organoid-S2	9,638	Unpublished	Query2
	Organoid-S3	5,748	Unpublished	Query2
CRC	GSE166555_12samples	66,050	TISCH2	Reference
	CRC-Normal (GSE139555_2samples)	10,112	TISCH2	Query1
	GSE146771_10samples[6]	43,817	TISCH2	Query2
GBC	GBC-Tumor-ref	11,629	Unpublished	Reference
	GBC-Normal-ref	2,607	Unpublished	Reference
	GBC-Tumor-query	3,100	Unpublished	Query1
	GBC-Normal-query	152	Unpublished	Query1
HCC	HCC-Tumor-ref1	9,740	Unpublished	Reference
	HCC-Tumor-ref2	6,066	Unpublished	Reference
	HCC-Tumor-ref3	3,485	Unpublished	Reference
	HCC-Tumor-ref4	19,180	Unpublished	Reference
	HCC-Tumor-ref5	6,924	Unpublished	Reference
	HCC-Normal-ref1	7,147	Unpublished	Reference
	HCC-Normal-ref2	4,905	Unpublished	Reference
	HCC-Tumor-1	1,609	Unpublished	Query1
	HCC-Tumor-2	14,486	Unpublished	Query2
	HCC-Tumor-S1	2,824	Unpublished	Query1
	HCC-Tumor-S2	1,799	Unpublished	Query2
	HCC-Normal	5,171	Unpublished	Query1
KC	KC-Tumor-ref1	7,973	Unpublished	Reference
	KC-Tumor-ref2	4,632	Unpublished	Reference

[2]ALL, acute lymphoblastic leukemia; AML, acute myeloid leukemia; BRCA, breast cancer; CHOL, cholangiocarcinoma; CRC, colorectal cancer; GBC, gallbladder cancer; HCC, hepatocellular carcinoma; KC, kidney cancer; LUAD, lung adenocarcinoma; NSCLC, non-small-cell lung cancer; PAAD, pancreatic adenocarcinoma; PBMC, peripheral blood mononuclear cell.

[3]There are 175 samples in total.

[4]There are 663,857 cells in total.

[5]In the column "Property", "Reference" and "Query1" were used for the construction of the reference dataset. The former was treated as the training set, while the latter was treated as the validation set. Then, "Reference" and "Query1" were both used for model training and identified malignant cells in test set "Query2".

[6]This dataset was also used in the cell subtype annotation module. Repeated calculations have been avoided when calculating the total number of cells and the total number of samples.

	KC-Tumor	10,276	Unpublished	Query1
LUAD	LUAD-Tumor-1	3,879	Unpublished	Query2
	LUAD-Tumor-2	1,055	Unpublished	Reference
	LUAD-Tumor-S1	2,271	Unpublished	Query2
LUAD(NSCLC)	NSCLC-Tumor-1 (GSE117570_4samples)	11,453	TISCH2	Query1
	NSCLC-Tumor-2 (GSE143423_3samples)	12,193	TISCH2	Reference
PAAD	PAC-Tumor-ref1	1,996	Unpublished	Reference
	PAC-Tumor-ref2	5,215	Unpublished	Reference
	PAC-Tumor-ref3	2,749	Unpublished	Reference
	PAC-Tumor-ref4	4,807	Unpublished	Reference
	GSE141017_1sample	4,917	TISCH2	Reference
	PAC-Normal-ref1	7,485	Unpublished	Reference
	PAC-Normal-ref2	5,251	Unpublished	Reference
	PAC-Normal-ref3	4,163	Unpublished	Reference
	PAC-Tumor	2,538	Unpublished	Query2
	PAC-Tumor-S1	3,393	Unpublished	Query1
	PAC-Tumor-S2	1,799	Unpublished	Query2
	PAC-Normal	4,890	Unpublished	Query1
	PAC-Normal-S1	7,766	Unpublished	Query2
	PAC-Normal-S2	4,034	Unpublished	Query2
	CRA001160_35samples	57,530	CRA001160	Query2
PBMC (normal)	PBMC_30K_10X	29,079	TISCH2	Reference
	PBMC_8K_10X	8,488	TISCH2	Query1

Table S5: The list of TCGA datasets used in malignant cell identification

Cancer type[7]	No. of samples	No. of malignant samples[8]	No. of normal samples[9]
BRCA	1222	1109	113
KIRC	611	539	72
LUAD	594	535	59
LUSC	551	502	49
PRAD	551	499	52
HNSC	546	502	44
COAD	521	480	41
SKCM	472	471	1
BLCA	433	414	19
LIHC	424	374	50
STAD	407	375	32
KIRP	321	289	32
PAAD	182	178	4
READ	177	167	10

[7]Refer to the cancer genome atlas program (TCGA) for the full name of cancer types.

[8]The tissue types named 'Additional New Primary', 'Additional Metastatic', 'Metastatic', 'Primary Tumor', or 'Recurrent Tumor' were labeled as "malignant".

[9]The tissue type named 'Solid Tissue Normal' was labeled as "nonMalignant".

Table S6: The list of spatial transcriptomics datasets used in scCancer2

Tissue types	No. of samples[10]	Sources[11]
Liver	28	Wu et al. (2021a)[12], Wu et al. (2022b)[13]
Breast	13	Wu et al. (2021b), 10X Genomics[14]
Bladder	4	Gouin et al. (2021)
Colorectal	6	Wu et al. (2022b), 10X Genomics
Ovarian	3	10X Genomics
Lung	2	10X Genomics
Prostate	2	10X Genomics
Cervical	1	10X Genomics
Intestinal	1	10X Genomics

[10] There are 60 samples in total.
[11] Access to 3 datasets in **Fig. 5** are listed below:
[12] http://lifeome.net/supp/livercancer-st/data.htm
[13] http://www.cancerdiversity.asia/scCRLM/
[14] https://www.10xgenomics.com/resources/datasets/
